# Chiral Pyridine-3,5-bis- (L-phenylalaninyl-L-leucinyl) Schiff Base Peptides as Potential Anticancer Agents: Design, Synthesis, and Molecular Docking Studies Targeting Lactate Dehydrogenase-A

**DOI:** 10.3390/molecules25051096

**Published:** 2020-02-29

**Authors:** Abd El-Galil E. Amr, Randa E. Abdel Mageid, Mohamed El-Naggar, Ahmed M. Naglah, Eman S. Nossier, Elsayed A. Elsayed

**Affiliations:** 1 Drug Exploration & Development Chair (DEDC), Pharmaceutical Chemistry Department, College of Pharmacy, King Saud University, Riyadh 11451, Saudi Arabia; aamr@ksu.edu.sa (A.E.-G.E.A.);; 2 Applied Organic Chemistry Department, National Research Center, Cairo, Dokki 12622, Egypt; 3 Photochemistry Department, National Research Center, Cairo, Dokki 12622, Egypt; randaabdelmagid@yahoo.com; 4 Chemistry Department, Faculty of Sciences, University of Sharjah, Sharjah 27272, UAE; m5elnaggar@yahoo.com; 5 Department of Peptide Chemistry, National Research Centre, Cairo 12622, Egypt; 6 Pharmaceutical Medicinal Chemistry Department, Faculty of Pharmacy (Girls), Al-Azhar University, Cairo 12622, Egypt; dr.emannossier@gmail.com; 7 Zoology Department, Bioproducts Research Chair, Faculty of Science, King Saud University, Riyadh 11451, Saudi Arabia; 8 Chemistry of Natural and Microbial Products Department, National Research Centre, Cairo, Dokki 12622, Egypt

**Keywords:** amino acids, tetrapeptides, Schiff bases, anticancer evaluation, p53 ubiquitination, LDHA, molecular docking

## Abstract

A series of branched tetrapeptide Schiff bases **3**–**6** were designed and synthesized from corresponding tetrapeptide hydrazide **2** as a starting material.In vitroevaluation of the synthesized compounds **4**–**6** against breast MCF-7 carcinoma cells identified their excellent anticancer potency, with IC_50_ ranging from 8.12 ± 0.14 to 17.55 ± 0.27 μM in comparison with the references, cisplatin and milaplatin (IC_50_= 13.34 ± 0.11and 18.43 ± 0.13 μM, respectively). Furthermore, all derivatives demonstrated promising activity upon evaluation of theirin vitroandin vivosuppression of p53 ubiquitination and inhibition assessment for LDHA kinase. Finally, molecular docking studies were performed to predict the possible binding features of the potent derivatives within the ATP pocket of LDHA in an attempt to get a lead for developing a more potent LDHA inhibitor with anti-proliferative potency.

## 1. Introduction

Peptides are short linear chains of amino acids (usually less than 50 AA in length) and are often stabilized by disulfide bonds [1]. Recently, there has been a noticeable increase in the number of publications covering the potential effects of peptides on lipid metabolism and blood pressure (BP), in addition to their antimicrobial, anti-inflammatory, analgesic, antioxidant, immunomodulatory, and anticancer activities [2,3,4].

Still, cancer is one of the most prominent causes of death in developed countries and there is a great demand for the discovery of new chemotherapies with more selectivity for cancer cells and less side effects [4,5]. The anticancer effects of peptides have been extensively explored with a raised number of approved peptide-based drugs [6,7,8]. The great potential of their antitumor activity has been exhibited through the action on multiple molecular pathways like enzymes involved in carcinogenesis with no genotoxicity [9].

Moreover, tumor-targeting peptides are effective alternative entities for the selective delivery of high doses of chemotherapeutic drugs or diagnostic agents to tumor sites while sparing normal tissues. Several peptide hormones have already been applied for tumor targeting, for example, the cyclic octapeptide analogue of somatostatin, octreotide, has been utilized for radiotargeting in neuroendocrine tumor [10]. Additionally, the linear peptide analogue of Luteinizing hormone-releasing hormone (LHRH), AN-152, has been used to target the LHRH receptor of breast cancer, ovarian cancer, and prostate cancer [11]. Recently, most peptidopyridine derivatives that have been synthesized consist of a wide range of pharmaceutical activities, including anticancer, analgesic, anti-convulsant, antiparkinsonian, analgesic, anti-inflammatory, and antimicrobial activities [12,13,14,15].

Schiff bases have been important candidates owing to their coordination chemistry, and they can be synthesized and used as ligands in building complex with different metal ions. On the other hand, Schiff bases have been interested with a broad spectrum of biological and pharmacological activities, such as antibacterial, DNA binding, anti HIV, Largicidal, antifertility, anticancer, and anti-inflammatory activities [16,17,18,19,20,21,22,23]. The broad-spectrum biological activities of Schiff bases have been attributed to the -C = N- imine bond, where the electrophilic carbon and nucleophilic nitrogen provide excellent binding characters with different nucleophiles and electrophiles, resulting in the inhibition of targeted diseases, enzymes, or DNA replication [24].

**Figure 1:**
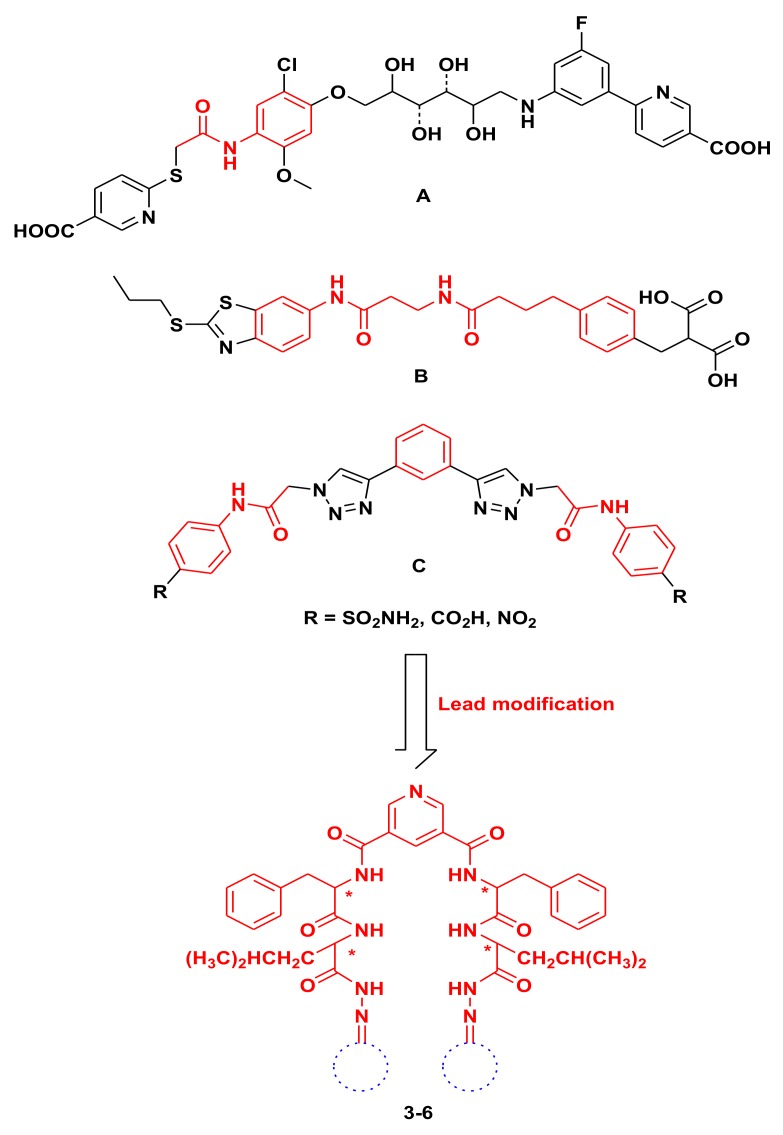
Design of new target derivatives **3**–**6** via lead modification of known candidates **A**–**C** having promising cytotoxic and LDHA inhibitory activities.

The lactate dehydrogenase (LDH) is one of the most prominent proteins and is responsible for the interconversion of lactate and pyruvate, parallel with the interconversion of NAD^+^ and NADH. LDH is present in humans as three subunits—LDHA, LDHB, and LDHC [25]. It is clear that cancer cells mainly produce energy through glycolysis and LDHA is the final enzyme involved in this pathway [26]. As LDHA is a vital supporter of glucose metabolism in cancer cells and its increased level is a marker for many tumors, LDHA can be defined as an anticancer drug target [27]. Previous literatures reported that V-shape structures illustrate good LDHA inhibition, so molecular modification of the potentially identified LDHA inhibitors **A**–**C** (Figure 1) might provide more effective hits [28,29,30].

In the present investigation and as a continuation of our previous work [31,32,33,34,35,36,37,38] in peptide and heterocyclic chemistry, chiral N3,N5-bis[(l-phenylalaninyl-l-leucinyl) substituted aryl or heterocyclic Schiff base]pyridine candidates were successfully designed, synthesized, and plausibly characterized by analytical techniques including IR, nuclear magnetic resonance (NMR), and mass spectroscopy evidences. In addition, their cytotoxicity effects on MCF-7 cancer cell lines, *in vivo* and *in vitro* inhibition of p53 ubiquitination, and lactate dehydrogenase-A were demonstrated in detail. Furthermore, molecular docking illustrates the binding affinity to LDHA kinase, which could facilitate the discovery of novel anticancer and LDHA inhibitory agents.

## 2. Results and Discussion

### 2.1. Chemistry

**Scheme 1:**
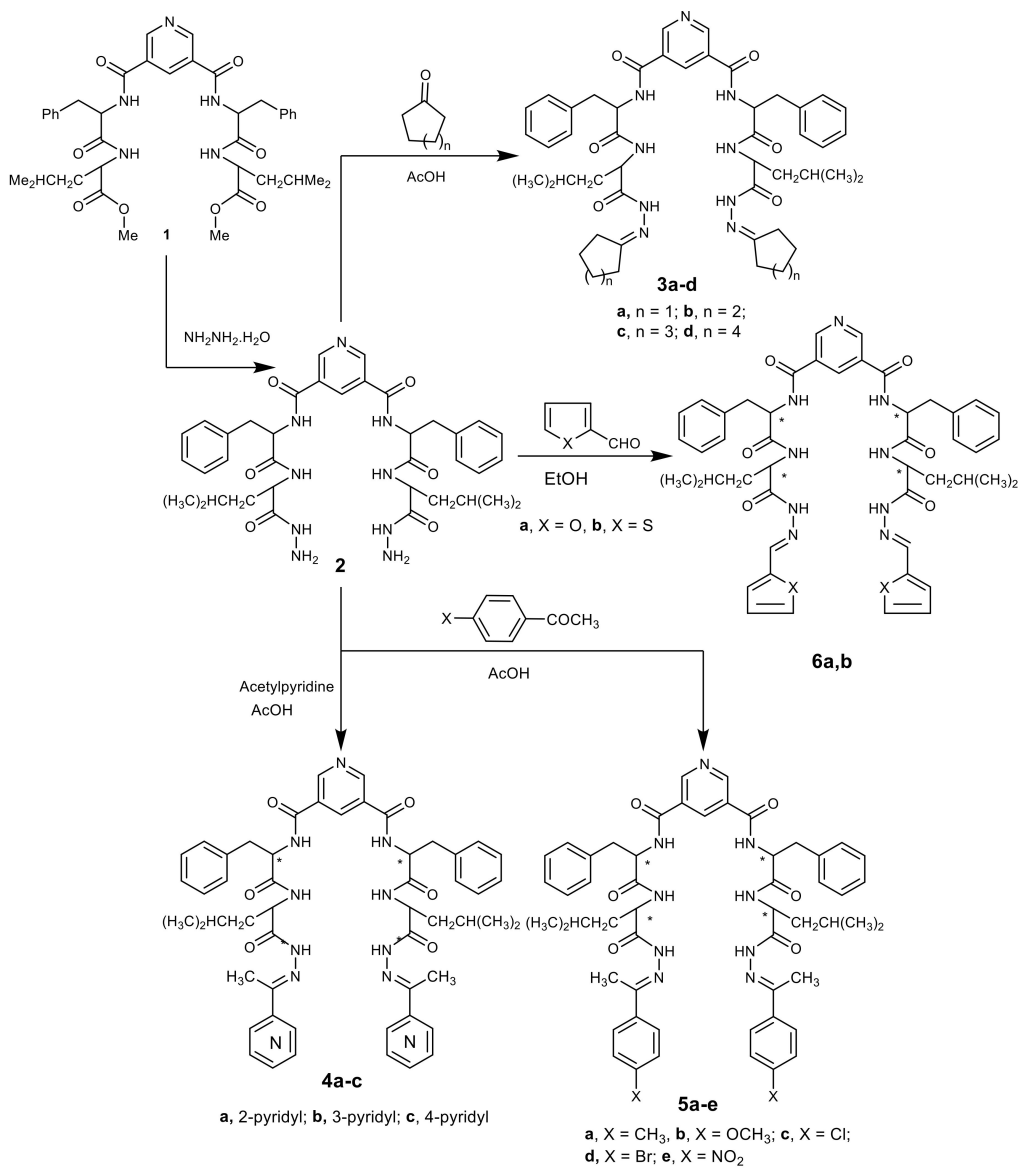
Synthetic routes for compounds **3a**–**d, 4a**–**c, 5a**–**e,** and **6a,b.**

In the present work, some of the prepared tetrapeptide derivatives **3**–**6** were obtained from *N^α^*-dipicolinoyl-bis[(l-phenylalanyl-l-leucine)hydrazide] **2**, which was obtained from corresponding ester **1** [39]. Reaction of hydrazide **2** with cycloalkanone derivatives in refluxing glacial acetic acid afforded the corresponding *N^α^*-dipicolinoyl-bis[(l-phenylalanyl-l-leucine)- N`-cycloalkylidenehydrazide] **3a**–**d**. Condensation of **2** with active carbonyl derivatives, namely, acetylpyridine or substituted acetophenone derivatives in refluxing acetic acid, gave the corresponding *N^α^*-dipicolinoyl-bis[(l-phenylalanyl-l-leucine)-N`-(1-(pyridyl)ethylidene)benzo- hydrazide] **4a**–**c** and *N^α^*-dipicolinoyl-bis-[(l-phenylalanyl-l-leucine)-N`-(1-(substituted phenyl) ethylidene)benzohydrazide] **5a**–**e**, respectively. Finally, treatment of heterocyclic aldehydes in refluxing ethanol gave the corresponding *N^α^*-dipicolinoylbis-[(l-phenylalanyl-l-leucine)-N`-(1- (furyl- **6a** and thienyl)ethylidene)benzohydrazide] **6b**, respectvely (Scheme 1).

### 2.2. Biological Evaluation

#### 2.2.1. Evaluation of In vitro Anticancer Potentials Against Breast Cancer

**Figure 2:**
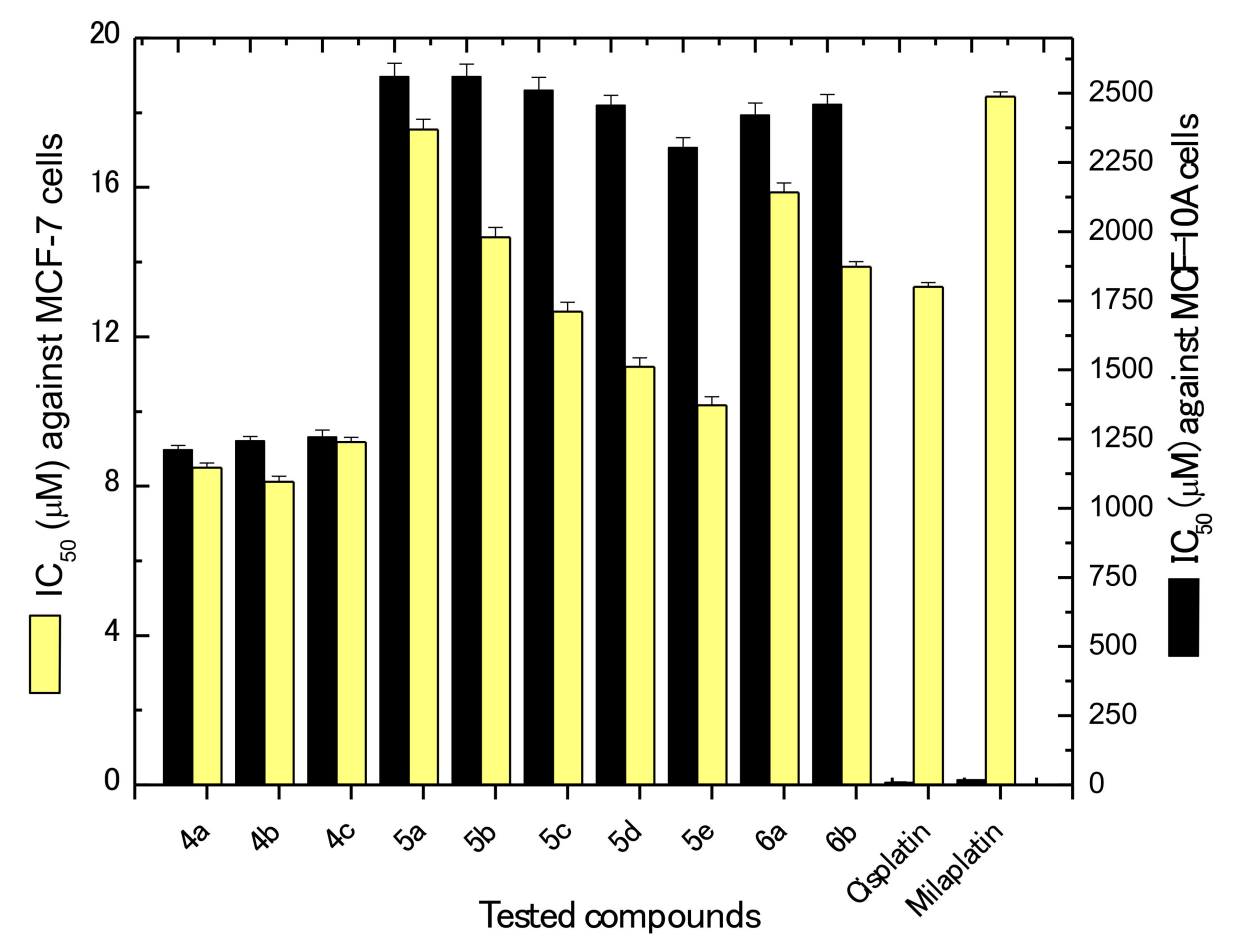
In vitro cytotoxic activities of the tested compounds **4**–**6** against MCF-7 and MCF-10A cell lines.

Following the synthesis of the new bis-dipeptide derivatives, we evaluated the anticancer potentials of these derivatives in vitro against breast cancer cells (MCF-7) as well as normal non tumorigenic MCF-10A cells. The obtained results were expressed as IC_50_ values in µM and were also compared for both cell lines with positive reference controls; that is cisplatin and milaplatin. The obtained results are presented in Figure 2. Generally, it can be seen that our newly synthesized bis-dipeptides have potential anticancer activities against MCF-7 cells. Furthermore, the obtained IC_50_ values ranged from 8.12 ± 0.14 to 17.55 ± 0.27 µM for compounds **4b** and **5a**, respectively. Additionally, all compounds were much active than milaplatin (IC_50_ = 18.43 ± 0.13 µM). Similarly, except for **5a**, **5b**, and **6a**, all other compounds were more active than cisplatin (IC_50_ = 13.34 ± 0.11 µM). The most active compound (**4b**) was more active by about 39.1% and 55.9% than cisplatin and milaplatin, respectively.

Generally, the anticancer activities of the prepared compounds can be arranged in descending order as follows: **4b > 4a> 4c > 5e > 5d > 5c > 6b > 5b>6a > 5a**. This arrangement can be related with the structure function relationship. Pyridine containing derivatives **4a**–**c** were more active than benzene ring-containing ones (**5a**–**d**), which were in turn less active than the five-membered ring compounds (**6a,b**). Furthermore, the nitrogen-position in pyridine nucleus affected the activities where the 3-pyridyl >2-pyridyl >4-pyridyl ones. This may be attributed to the aromaticity and the location of the nitrogen atom in the pyridine ring. On the other hand, substitution on the benzene ring affected the anticancer activity (nitro **5e** > bromo **5d** > chloro **5c** > methoxyl **5b** > methyl **5a**), owing to the difference in the electron withdrawing or donation capacity of the substituting group. Finally, the substitution with five-membered rings afforded notably increased activity in the thiophene derivative **6b** than the furan one **6a**, owing to the difference in electronegativity between the oxygen and sulfur atoms. Moreover, the larger size of the sulfur atom makes the thiophene ring more similar in size to a phenyl ring and gives it a more pronounced aromatic character, compared with the furan ring, thus more closely resembling the benzene ring of the active compounds. Bis-dipeptide Schiff bases have been previously reported to exhibit anticancer activities owing to their ionophoric properties, which may contribute to apoptosis induction [40,41].

It is worth mentioning that comparing IC_50_results between both MCF-7 and non-tumorigenic MCF-10A cells revealed that our newly synthesized compounds are far less toxic against normal cells. The IC_50_ values obtained for the most and least potent derivatives (**4b** and **5a**, respectively) were higher by about 142.7- and 145.9-fold, respectively, against MCF-10A. It is generally preferable that pharmaceutical candidates with potential anticancer properties should exhibit less toxicity against normal cells. The fact that normal cells are usually less affected by possible anticancer candidates may be attributed to the fact that, upon cancer initiation and development, cancer cells overcome normal growth regulation pathways present in normal cells, and thusthey tend to be affected by other different regulatory growth pathways, which are not evident in the case of normal cells.

#### 2.2.2. In vivo Anti-Breast Cancer

**Figure 3:**
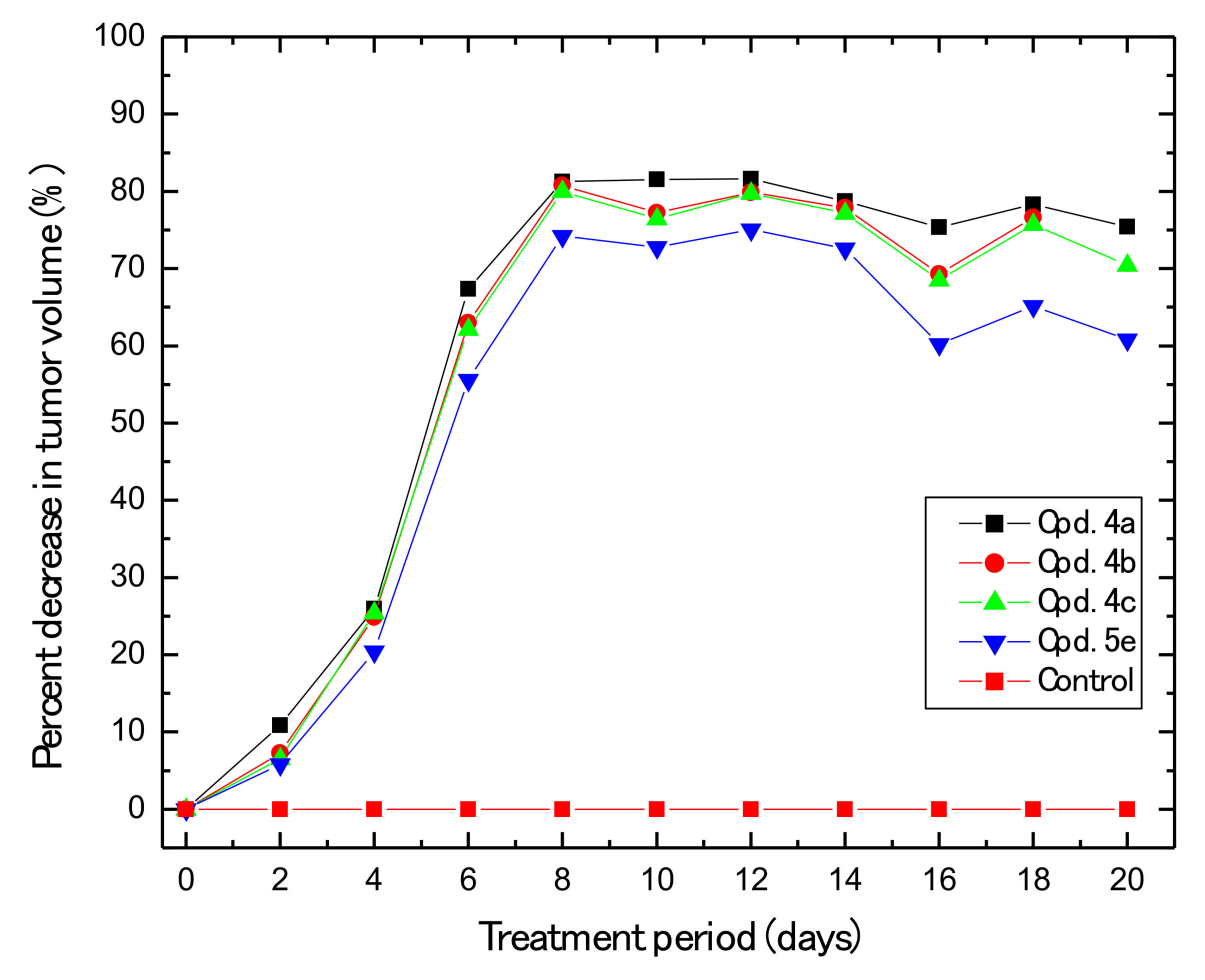
Decreased percentage ofin vivotumor growth in response to different compound treatments.

In this section, we developed a breast cancer animal xenograft model for the evaluation of thein vivo anticancer potential of our newly prepared compounds. We chose the derivatives that showed the lowest IC_50_ values in the previous in vitro investigation; namely, **4a, 4b, 4c**, and **5e**. The results obtained (Figure 3) showed that all four tested compounds have a great potential to decrease tumor growth in animal model, where the decrease in tumor growth effect increased gradually with the treatment period up to eight days of treatment. At that point, the highest decrease in tumor growth obtained ranged from 81.3% to 74.2% for compounds **4a** and **5e**, respectively. After wards, the noticeable effects were more or less constant up to 14 days of treatment. However, by the end of the treatment period (20 days), the percentage of tumor growth inhibition decreased to about 75.4%, 73.1%, 70.5%, and 60.8% for compounds **4a**, **4a**, **4c**, and **5e**, respectively. Our results are in good agreement with those previously published on the *in vivo* anticancer potential of Schiff bases owing to apoptosis induction [42].

#### 2.2.3. In vitro and In vivo Inhibition of p53 

Several cancer prevention drugs depend mainly on the ubiquitination activities against p53 as a suppressor protein. When E3 ubiquitin protein ligase HDM2 binds to p53, the resulting mechanism is the inhibition of its ability as a transcription activator. Accordingly, the regulative mechanism is acting negatively. Therefore, blocking the p53 binding site on HDM2 provides for a better possible antitumorigenic molecule. Currently, murine double minute 2 (MDM2) is considered as the regulator of choice when it comes to investigatingp53 inhibition [43].

**Figure 4:**
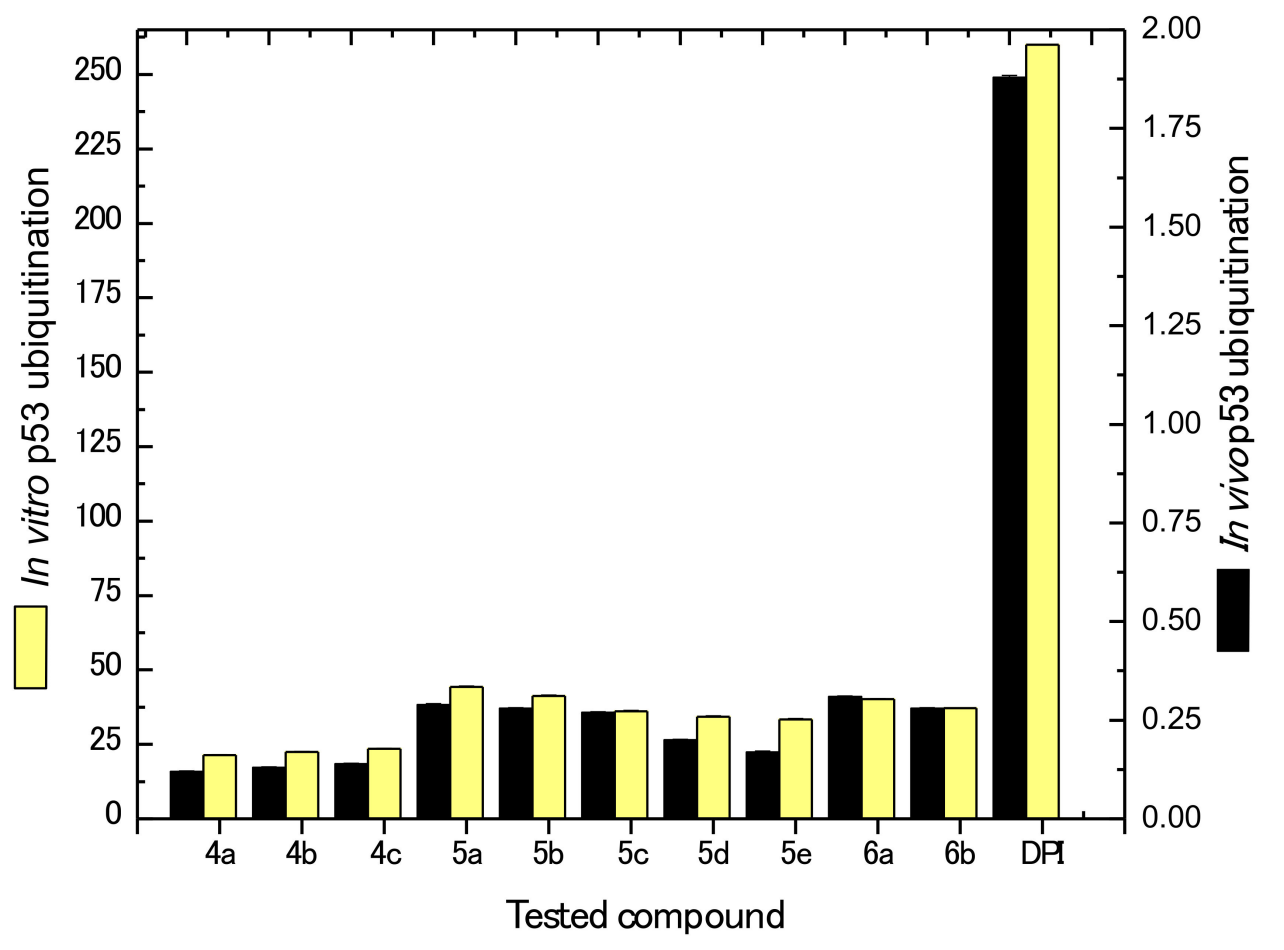
In vitro and in vivop53 ubiquitination of the newly synthesized compounds.

The results obtained (Figure 4) showed that all prepared derivatives exhibited potential in vitro as well as in vivo suppression of p53 ubiquitination when compared with the positive control (diphenyl imidazole). The obtained in vitro IC_50_values ranged from 21.34 ± 0.13 to 44.33 ± 0.34 µM for compounds **4a** and **5a**, respectively. For thein vivo results, the IC_50_ values ranged from 0.12 ± 0.0011 to 0.31 ± 0.0010 µM for compounds **4a** and **6a**, respectively. Furthermore, it can be seen that inhibitory effects of **4a** were higher by about 12.2- and 15.7-fold compared with the positive control for in vitro and in vivo p53 ubiquitination, respectively.

#### 2.2.4. Kinase Inhibition Studies

**Figure 5:**
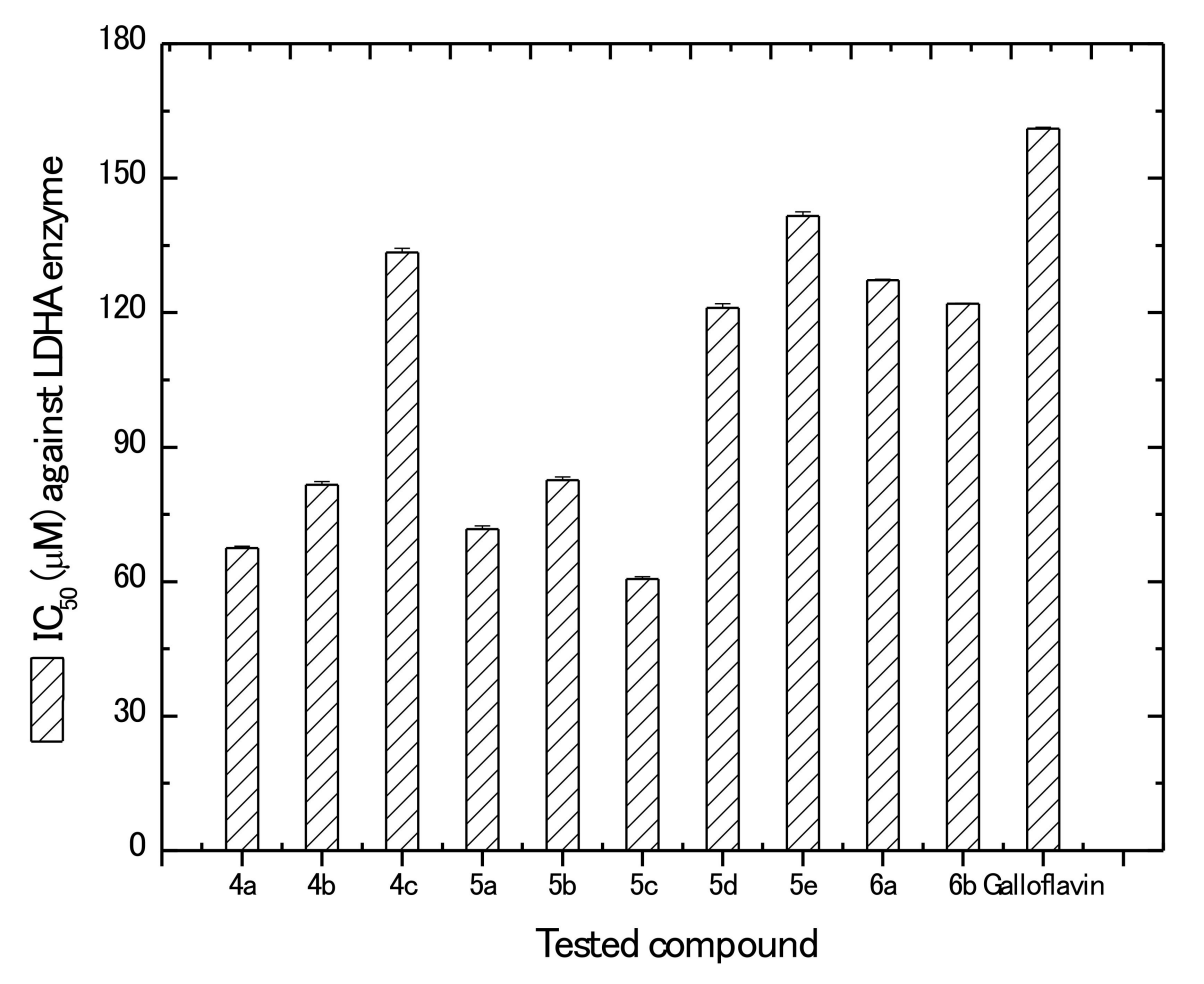
In vitro kinase inhibition activity of the tested compounds **4**–**6** against human lactate dehydrogenase-A.

The newly synthesized compounds **4**–**6** were evaluated for their ability to inhibit LDHA in comparison with galloflavin as a known isoform nonselective LDHA inhibitor [44,45]. The choice of galloflavin as a reference was based on the fact that it is potent anticancer agent and commercially available [46,47,48,49,50]. The results presented in Figure 5 show that all investigated derivatives exhibited better LDHA inhibition compared with galloflalvin, where the IC_50_ values ranged from 60.54 ± 0.56 to 141.56 ± 0.98 µM for compounds **5c** and **5e**, respectively. These values correspond to about 2.66- and 1.14-fold in increase in the inhibitory activity compared with that recorded for galloflavin IC_50_ = 161.05 ± 0.32 µM).

### 2.3. Molecular Modeling Studies

**Figure 6:**
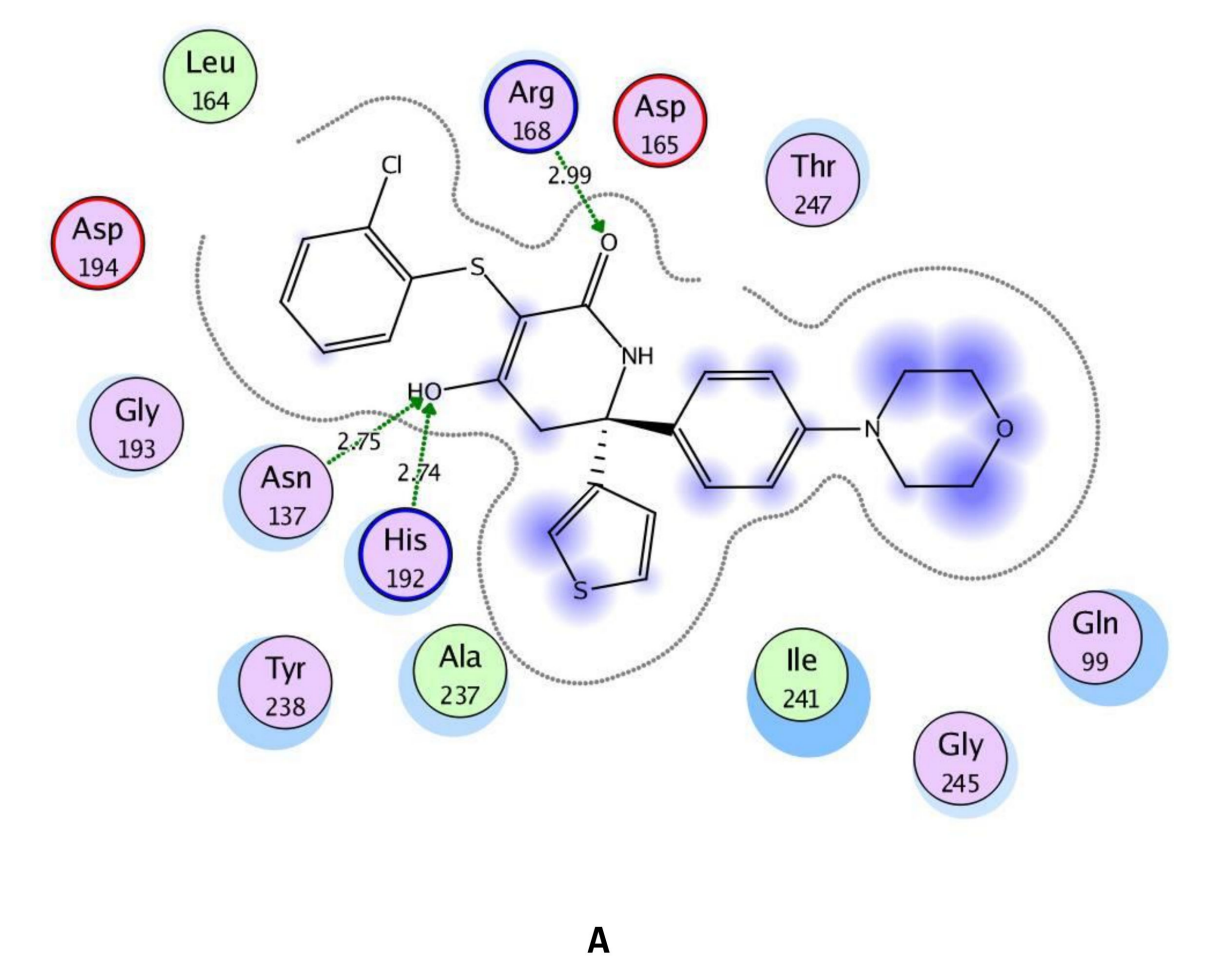

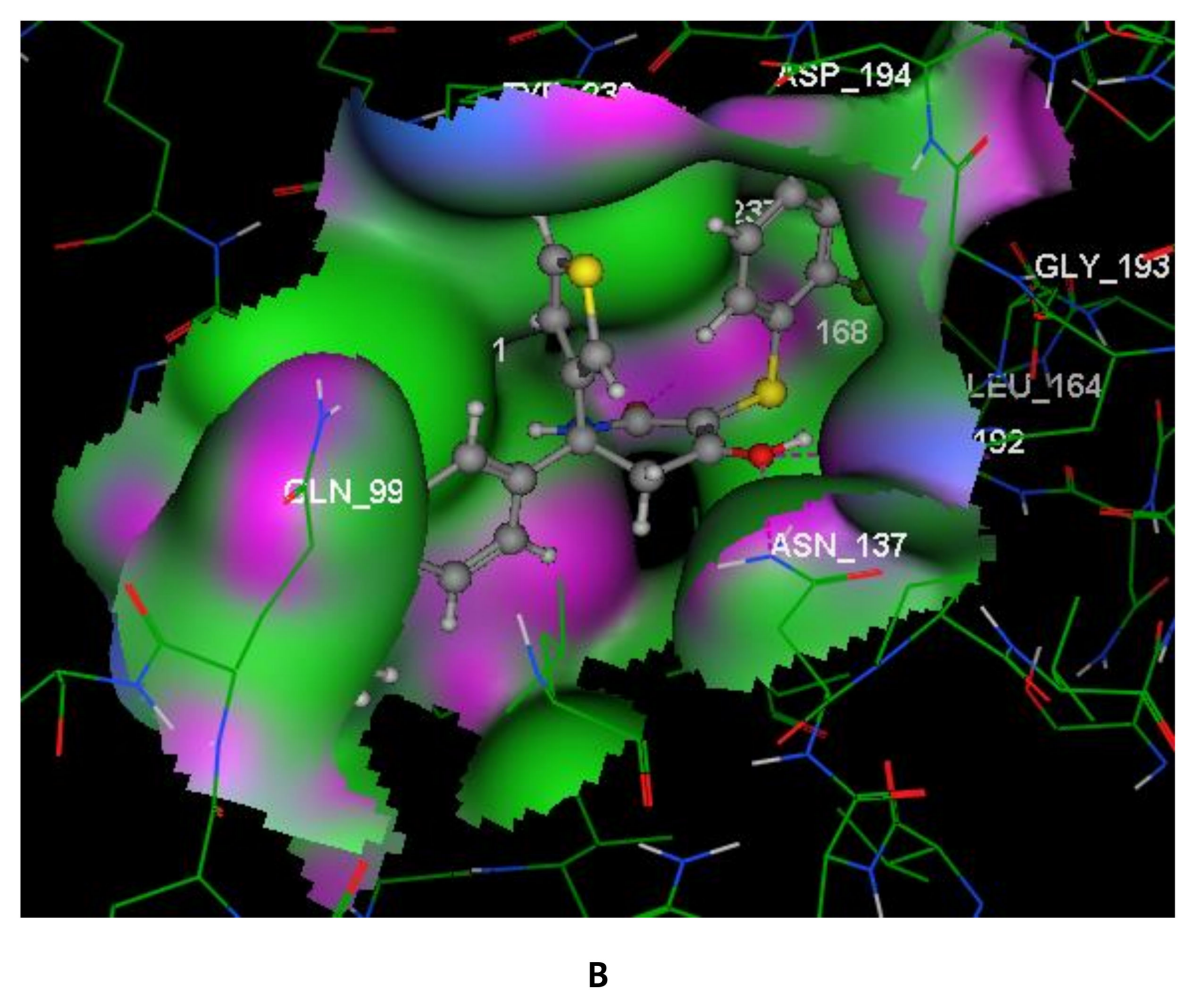
Two-dimensional (**A**) and 3D (**B**) patternsillustrating the proposed binding mode of the co-crystallized ligand GNE-140 in the LDHA binding pocket (PDB code: 4ZVV). Hydrogen bonding interactions with the protein are shown as dashed lines. Green color reflects a hydrophobic area, pink color reflects a high polar area, and blue color reflects a mild polar area.

**Figure 7:**
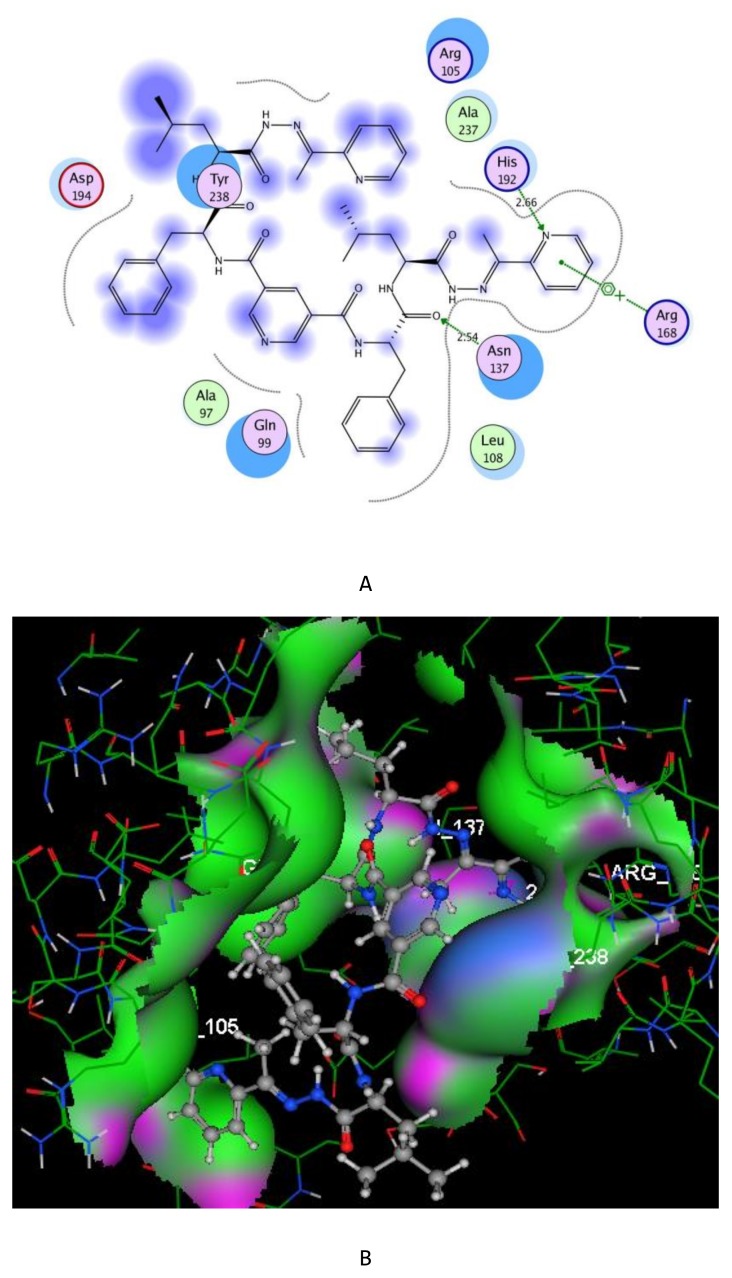
Two-dimensional (**A**) and 3D (**B**) patternsillustrating the proposed binding mode of the compound **4a** in the LDHA binding pocket (PDB code: 4ZVV). Hydrogen bonding interactions with the protein are shown as dashed lines. Green color reflects a hydrophobic area, pink color reflects a high polar area, and blue color reflects a mild polar area.

**Figure 8:**
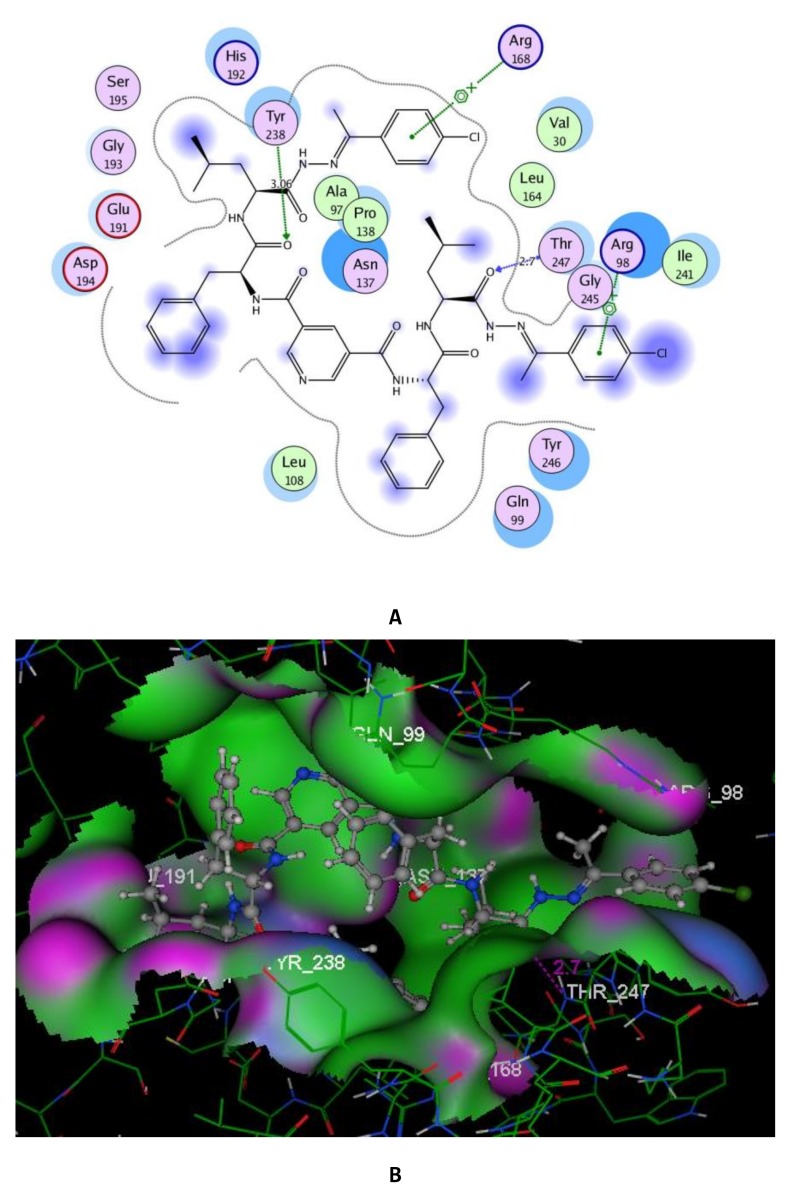
Two-dimensional (**A**) and 3D (**B**) patternsillustrating the proposed binding mode of the compound **5c** in the LDHA binding pocket (PDB code: 4ZVV). Hydrogen bonding interactions with the protein are shown as dashed lines. Green color reflects a hydrophobic area, pink color reflects a high polar area, and blue color reflects a mild polar area.

In order to further understand and illustrate the interaction of compounds **4**–**6** with the LDHA active site, the docking modeling studies were done in an open loop conformation of the enzyme. The docking studies were developed using Molecular Operating Environment software, MOE 10.2008 software [51,52] with X-ray crystallographic structure of LDHA (PDB ID: 4ZVV) [53]. The root mean square deviation (RMSD) value of 8.93 was calculated via redocking of the co-crystallized ligand, GNE-140, into the pocket site of LDH-A with docking score energy of −9.73 kcal/mol. The docking scores of the screened compounds **4**–**6** were all in the range of −15.60 to −11.29 kcal/mol. Representations of the docking results of these compounds with LDHA are depicted in Table 1and Figure 6, Figure 7 and Figure 8.

As the reported binding mode of GNE-140 with LDHA [53], the oxygen of the hydroxyl group binds to the active site via two hydrogen bond acceptors with the sidechains of **Asn137** and **His192** (distance: 2.75 and 2.74 Å), respectively. Additionally, the carbonyl oxygen forms H-bonding with the sidechain of **Arg168** (distance: 2.99 Å) (Figure 6).

The increased inhibitory activity of the newly synthesized derivatives **4–6** against LDHA enzyme could be partially explained by the evidence obtained from the docking studies (Table 1). By focusing on the highly potent inhibitors **4a** and **5c** (Figure 7 and Figure 8), the essential residue **Arg168** connected to the newly formed 2-pyridyl (in **4a**) and 4-chlorophenyl moieties (in **5c**) by arene–cation interactions. Moreover, the compound **4a** was fitted nicely in the active pocket through two hydrogen bond interactions between nitrogen of 2-pyridyl moiety and oxygen of the amide group with the key amino acids **His192** and **Asn137** (distance: 2.66 and 2.54 Å), respectively.

The enhanced inhibitory potency of **5c** may be attributed to its stabilization within the ATP binding pocket via further arene–cation interaction between the other 4-chlorophenyl and **Arg98**, and two H-bond acceptors between the oxygens of the two amide groups located at opposite sites around the parent pyridine ring with the sidechain of **Tyr238** and the backbone of **Thr247** (distance: 3.06 and 2.70 Å), respectively. Additionally, the essential amino acids **Ala97**, **Asn137**, and **Pro138** were located between the two wings of the symmetrical side chains facing the central core (pyridine moiety) (Figure 8).

## 3. Materials and Methods 

### 3.1. Chemistry

Melting points were determined in open glass capillary tubes with an electro thermal digital melting point apparatus (model: IA9100), and are uncorrected. Elemental microanalysis for carbon, hydrogen, and nitrogen (Microanalytical Unit, NRC) was found within the acceptable limits of the calculated values. Infrared spectra (KBr) were recorded on a Nexus 670 FTIR Nicolet, Fourier Transform infrared spectrometer. Proton and carbon nuclear magnetic resonance (^1^H- and ^13^C NMR) spectra were run in (DMSO-d_6_) on Jeol 500 MHz instruments. Mass spectra were run on a MAT Finnigan SSQ 7000 spectrometer, using the electron impact technique (EI). Analytical thin layer chromatography (TLC) was performed on silica gel aluminum sheets, 60 F_254_ (E. Merck). Compound **2** was prepared according to our previous reported procedure [39].

**Synthesis of N^α^-dinicotinoyl-bis[L-phenylalaninyl-L-leucyl]cycloalkanonehydrazones 3a**–**d.** A mixture of hydrazide **2** (1 mmol) and cycloalkanone, namely, cyclopentanone, cyclohexanone, cycloheptanone, or cyclooctanone (2 mmol), in glacial acetic acid (30 mL) was refluxed for 6 h. The reaction mixture was poured onto ice water; the formed precipitate was filtered off, dried, and crystallized from AcOH/H_2_O to give compounds **3a**–**d**, respectively.

**N^3^,N^5^-Bis{1-[(1-(2-cyclopentylidenehydrazinyl)-4-methyl-1-oxopentan-2-yl]amino)-1-oxo-3-phenylpropan-2-yl}pyridine-3,5-dicarboxamide (3a):** Yield 72%, mp 232–234 °C. [*α*]${}_{D}^{25}$ = –98 (*c* = 0.5, DMF). IR ν, cm^–1^: 3518-3334 (NH), 3090 (CH-Ar), 2985 (CH-aliph.), 1654, 1532, 1254 (C=O, amide I, II and III). ^1^H NMR spectrum (DMSO-d_6_), δ_H_, ppm:0.90–0.95 (m, 12H, 4CH_3_),1.24–1.35 (m, 16H, 8 CH_2_), 1.58–1.70 (t, 4H, 2CH_2_), 2.18–2.25 (m, 2H, 2CH), 3.42 (d, 4H, 2CH_2_), 4.35 (t, 2H, 2CH), 4.55 (t, 2H, 2CH), 6.95–7.65 (m, 10H, 2Ph-H), 8.38, 9.05 (2s, 3H, pyr-H), 8.65, 8.75, 9.15 (3s, 6H, 6NH, exchangeable with D_2_O). ^13^C NMR spectrum (DMSO-d_6_), δ_C_, ppm: 17.32, 17.54 (4C, 4CH_3_), 24.15, 29.42, 34.32, 186.86 (10C, cyclopentyl ring), 23.98 (2C, 2CH), 40.12 (2C, 2CH_2_), 42.26 (2C, 2CH_2_), 52.48, 53.12 (4C, 4 CH), 124.18, 128.26, 129.35, 138.64 (12C, 2Ph-C), 131.55, 140.13, 152.12 (5C, pyr-C), 163.72, 169.15 (4C, 4CO-amide), 174.40 (2C, 2CO-hydrazone). MS (EI, 70 eV): *m/z* (%) = 848 (45) [M]^+^. Found, %: C 66.45; H 7.18; N 14.76. C_47_H_61_N_9_O_6_ (848.04). Calculated, %: C 66.57; H 7.25; N 14.86.

N^3^,N^5^-Bis{1-[(1-(2-cyclohexylidenehydrazinyl)-4-methyl-1-oxopentan-2-yl)amino]-1-oxo-3-phenylpropan-2-yl}pyridine-3,5-dicarboxamide (3b): Yield 65%, mp 198–200 °C. [*α*]${}_{D}^{25}$ = –118 (*c* = 0.5, DMF). IR ν, cm^−1^: 3510–3387 (NH), 3088 (CH-Ar), 2989 (CH-aliph.), 1655, 1533, 1254 (C=O, amide I, II and III). ^1^H NMR spectrum (DMSO-d_6_), δ_H_, ppm:0.86–0.94 (m, 12H, 4CH_3_),1.50–1.65 (m, 16H, 8CH_2_), 2.22–2.26 (m, 2H, 2CH), 2.10–2.14 (m, 8H, 4CH_2_), 3.46 (d, 4H, 2CH_2_), 4.34 (t, 2H, 2CH), 4.54 (t, 2H, 2CH), 6.98–7.68 (m, 10H, 2Ph-H), 8.42, 9.09 (2s, 3H, pyr-H), 8.64, 8.78, 9.16 (3s, 6H, 6NH, exchangeable with D_2_O). ^13^C NMR spectrum (DMSO-d_6_), δ_C_, ppm: 17.30, 17.42 (4C, 4CH_3_), 24.76, 27.02, 28.32, 165.85 (12C, cyclohexyl ring), 23.90 (2C, 2CH), 40.18 (2C, 2CH_2_), 42.34 (2C, 2CH_2_), 52.45, 53.16 (4C, 4 CH), 124.24, 128.28, 129.38, 138.65 (12C, 2Ph-C), 131.58, 140.15, 152.16 (5C, pyr-C), 163.76, 169.18, 174.42 (6C, 6 CO-amide). MS (EI, 70 eV): *m/z* (%) = 876 (32) [M]^+^. Found, %: C 67.00; H 7.42; N 14.30. C_49_H_65_N_9_O_6_ (876.10). Calculated, %: C 67.18; H 7.48; N 14.39.

**N^3^,N^5^-Bis{1-[(1-(2-cycloheptylidenehydrazinyl)-4-methyl-1-oxopentan-2-yl)amino]-1-oxo-3-phenylpropan-2-yl}pyridine-3,5-dicarboxamide (3c):** Yield 56%, mp 221–223 °C. [*α*]${}_{D}^{25}$ = –96 (*c* = 0.5, DMF). IR ν, cm^−1^: 3412–3375 (NH), 3092 (CH-Ar), 2995 (CH-aliph.), 1655, 1535, 1255 (C=O, amide I, II and III). ^1^H NMR spectrum (DMSO-d_6_), δ_H_, ppm: ^1^H NMR spectrum (DMSO-d_6_), δ_H_, ppm: 0.88–0.96 (m, 12H, 4CH_3_), 1.50–1.70 (m, 20H, 10CH_2_), 2.20–2.24 (m, 2H, 2CH), 2.02–2.10 (m, 8H, 4CH_2_), 3.45 (d, 4H, 2CH_2_), 4.44 (t, 2H, 2CH), 4.58 (t, 2H, 2CH), 6.92-7.60 (m, 10H, 2Ph-H), 8.54, 9.00 (2s, 3H, pyr-H), 8.65, 8.76, 9.15 (3s, 6H, 6NH, exchangeable with D_2_O). ^13^C NMR spectrum (DMSO-d_6_), δ_C_, ppm: 17.40, 17.44 (4C, 4CH_3_), 27.12, 28.82, 30.28, 36.12, 183.90 (14C, cycloheptyl ring), 24.00 (2C, 2CH), 40.24 (2C, 2CH_2_), 42.46 (2C, 2CH_2_), 52.40, 53.24 (4C, 4 CH), 124.28, 128.30, 129.42, 138.64 (12C, 2Ph-C), 131.60, 140.18, 152.18 (5C, pyr-C), 163.72, 169.24, 174.40 (6C, 6 CO-amide). MS (EI, 70 eV): *m/z* (%) = 902 (14) [M-2]^+^. Found, %: C 67.64; H 7.61; N 13.86. C_51_H_69_N_9_O_6_ (904.15). Calculated, %: C 67.75; H 7.69; N 13.94.

**N^3^,N^5^-Bis{1-[(1-(2-cyclooctylidenehydrazinyl)-4-methyl-1-oxopentan-2-yl)amino]-1-oxo-3-phenylpropan-2-yl}pyridine-3,5-dicarboxamide (3d):** Yield 56%, mp 242–244 °C. [*α*]${}_{D}^{25}$ = –104 (*c* = 0.5, DMF). IR ν, cm^–1^: 3418–3360 (NH), 3086 (CH-Ar), 2990 (CH-aliph.), 1654, 1533, 1255 (C=O, amide I, II and III). ^1^H NMR spectrum (DMSO-d_6_), δ_H_, ppm:0.84–0.90 (m, 12H, 4CH_3_), 1.22–1.60 (m, 20H, 10CH_2_), 1.65–1.70 (m, 4H, 2CH_2_), 2.00–2.08 (m, 8H, 4CH_2_), 2.18–2.22 (m, 2H, 2CH), 3.52 (d, 4H, 2CH_2_), 4.20–4.35 (m, 2H, 2CH), 4.60–4.68 (m, 2H, 2CH), 6.96–7.54 (m, 10H, 2Ph-H), 8.46, 9.05 (2s, 3H, pyr-H), 8.68, 8.74, 9.12 (3s, 6H, 6NH, exchangeable with D_2_O). ^13^C NMR spectrum (DMSO-d_6_), δ_C_, ppm: 17.30, 17.54 (4C, 4 CH_3_), 22.92, 24.80, 25.28, 27.02, 28.79, 154.94 (16C, cyclooctyl ring), 23.68 (2C, 2CH), 40.10 (2C, 2CH_2_), 42.56 (2C, CH_2_), 52.62, 53.12 (4C, 4 CH), 124.24, 128.25, 129.40, 138.65 (12C, 2Ph-C), 131.70, 140.34, 152.25 (5C, pyr-C), 163.68, 169.16, 174.45 (6C, 6 CO-amide). MS (EI, 70 eV): *m/z* (%) = 932 (32) [M]^+^. Found, %: C 68.18; H 7.80; N 13.45. C_53_H_73_N_9_O_6_ (932.22). Calculated, %: C 68.29; H 7.89; N 13.52.

**Synthesis of N^α^-dinicotinoyl-bis[L-phenylalaninyl-L-leucyl] Schiff bases 4a–c, 5a–e, and 6a,b.** A mixture of **2** (1 mmol) and acetylpyridines or substituted acetophenones or heterocyclic aldehyde, namely, 2-, 3-, 4-acetylpyridine,4-methyl-, 4-methoxy-, 4-chloro-, 4-bromo-, 4-nitroacetophenone or 2-furane, 2-thiophene aldeydes (2 mmol) in AcOH or ethanol (30 mL) was refluxed for 4–6 h, and then evaporated to dryness. The formed residue was solidified with *n*-hexane, filtered off, and crystallized from the proper solvents to give Schiff base derivatives **4a–c, 5a–e,**and **6a,b,** respectively.

**N^3^,N^5^-Bis{1-[(4-methyl-1-oxo-1-(2-(1-(pyridin-2-yl)ethylidene)hydrazinyl)pentan-2-yl)amino]-1-oxo-3-phenylpropan-2-yl}pyridine-3,5-dicarboxamide (4a):** Yield 60%, mp 216–218 °C (DMF/H_2_O). [*α*]${}_{D}^{25}$ = −112 (*c* = 0.5, DMF). IR ν, cm^−1^: 3450–3354 (NH), 3092 (CH-Ar), 2986 (CH-aliph.), 1654, 1534, 1252 (C=O, amide I, II and III). ^1^H NMR spectrum (DMSO-d_6_), δ_H_, ppm: 0.85–0.95 (m, 12H, 4CH_3_), 1.60–1.70 (m, 4H, 2CH_2_), 1.82 (s, 6H, 2CH_3_), 2.20-2.30 (m, 2H, 2CH), 3.48 (d, 4H, 2CH_2_), 4.25–4.34 (m, 2H, 2CH), 4.60–4.70 (m, 2H, 2CH), 6.92–7.65 (m, 10H, 2Ph-H), 7.68–8.50 (m, 10H, pyr-H), 9.04 (s, 1H, pyr-H-4), 8.65, 8.85, 9.15 (3s, 6H, 6NH, exchangeable with D_2_O). ^13^C NMR spectrum (DMSO-d_6_), δ_C_, ppm: 13.45, 17.85, 18.00 (6C, 6 CH_3_), 23.96 (2C, 2CH), 40.56 (2C, 2CH_2_), 42.44 (2C, CH_2_), 52.56, 53.12 (4C, 4 CH), 124.24, 128.26, 129.35, 138.68 (12C, 2Ph-C), 145.30 (2C, 2C=N), 122.90, 126.00, 131.60, 135.80, 140.20, 148.62, 152.22, 154.48 (15C, 3 pyr-C), 163.82, 169.33 (4C, 4 CO-amide), 174.55 (2C, 2CO-hydrazone). MS (EI, 70 eV): *m/z* (%) = 922 (54) [M]^+^. Found, %: C 66.32; H 6.36; N 16.60. C_51_H_59_N_11_O_6_ (922.08). Calculated, %: C 66.43; H 6.45; N 16.71.

**N^3^,N^5^-Bis{1-[(4-methyl-1-oxo-1-(2-(1-(pyridin-3-yl)ethylidene)hydrazinyl)pentan-2-yl)-amino]-1-oxo-3-phenylpropan-2-yl}pyridine-3,5-dicarboxamide (4b):** Yield 58 %, mp 208–210 °C (AcOH/H_2_O). [*α*]${}_{D}^{25}$ = −132 (*c* = 0.5, DMF). IR ν, cm^−1^: 3467–3350 (NH), 3075 (CH-Ar), 2990 (CH-aliph.), 1652, 1536, 1252 (C=O, amide I, II and III). ^1^H NMR spectrum (DMSO-d_6_), δ_H_, ppm: 0.88-0.93 (m, 12H, 4CH_3_), 1.62–1.74 (m, 4H, 2CH_2_), 1.80 (s, 6H, 2CH_3_), 2.16–2.24 (m, 2H, 2CH), 3.45 (d, 4H, 2CH_2_), 4.24–4.35 (m, 2H, 2CH), 4.62-4.70 (m, 2H, 2CH), 6.96-7.60 (m, 10H, 2Ph-H), 7.72-8.65 (m, 8H, pyr-H), 9.04, 9.08 (3s, 3H, pyr-H), 8.70, 8.86, 9.18 (3s, 6H, 6NH, exchangeable with D_2_O). ^13^C NMR spectrum (DMSO-d_6_), δ_C_, ppm: 15.25, 17.88, 18.02 (6C, 6 CH_3_), 23.94 (2C, 2CH), 40.55 (2C, 2CH_2_), 42.45 (2C, CH_2_), 52.55, 53.15 (4C, 4 CH), 124.25, 128.25, 129.35, 138.65 (12C, 2Ph-C), 145.35 (2C, 2C=N), 122.96, 126.14, 131.65, 137.56, 140.45, 147.82, 151.32, 154.80 (15C, 3 pyr-C), 163.80, 169.14 (4C, 4CO-amide), 168.12 (2C, 2CO-hydrazone). MS (EI, 70 eV): *m/z* (%) = 921 (18) [M-1]^+^. Found, %: C 66.33; H 6.34; N 16.64. C_51_H_59_N_11_O_6_ (922.08). Calculated, %: C 66.43; H 6.45; N 16.71.

**N^3^,N^5^-Bis{1-[(4-methyl-1-oxo-1-(2-(1-(pyridin-4-yl)ethylidene)hydrazinyl)pentan-2-yl)-amino]-1-oxo-3-phenylpropan-2-yl}pyridine-3,5-dicarboxamide (4c):** Yield 75%, mp 196–198 °C (EtOH/H_2_O). [*α*]${}_{D}^{25}$ = −140 (*c* = 0.5, DMF). IR ν, cm^–1^: 3466–3348 (NH), 3080 (CH-Ar), 2986 (CH-aliph.), 1655, 1535, 1255 (C=O, amide I, II and III). ^1^H NMR spectrum (DMSO-d_6_), δ_H_, ppm: 0.86-0.90 (m, 12H, 4CH_3_), 1.65–1.70 (m, 4H, 2CH_2_), 1.96 (s, 6H, 2CH_3_), 2.18–2.20 (m, 2H, 2CH), 3.46 (d, 4H, 2CH_2_), 4.25–4.32 (m, 2H, 2CH), 4.60–4.68 (m, 2H, 2CH), 6.98–7.64 (m, 10H, 2Ph-H), 7.70–8.65 (m, 10H, pyr-H), 9.06 (s, 1H, pyr-H), 8.75, 8.85, 9.14 (3s, 6H, 6NH, exchangeable with D_2_O). ^13^C NMR spectrum (DMSO-d_6_), δ_C_, ppm: 16.02, 17.86, 18.12 (6C, 6 CH_3_), 23.86 (2C, 2CH), 40.50 (2C, 2CH_2_), 42.60 (2C, CH_2_), 52.54, 53.18 (4C, 4 CH), 124.60, 128.42, 129.38, 138.658 (12C, 2Ph-C), 146.30 (2C, 2C=N), 124.14, 131.60, 138.50, 140.40, 147.50, 149.54 (15C, 3 pyr-C), 164.15, 169.46 (4C, 4CO-amide), 174.10 (2C, 2CO-hydrazone). MS (EI, 70 eV): *m/z* (%) = 920 (8) [M-2]^+^. Found, %: C 66.37; H 6.35; N 16.65. C_51_H_59_N_11_O_6_ (922.08). Calculated, %: C 66.43; H 6.45; N 16.71.

**N^3^,N^5^-Bis{1-[(1-(2-(1-(4-methoxyphenyl)ethylidene)hydrazinyl)-4-methyl-1-oxopentan-2-yl)-amino]-1-oxo-3-phenylpropan-2-yl}pyridine-3,5-dicarboxamide (5a):** Yield 55%, mp 202–204 °C (AcOH/H_2_O). [*α*]${}_{D}^{25}$ = −110 (*c* = 0.5, DMF). IR ν, cm^−1^: 3478–3348 (NH), 3085 (CH-Ar), 2976 (CH-aliph.), 1655, 1534, 1253 (C=O, amide I, II and III). ^1^H NMR spectrum (DMSO-d_6_), δ_H_, ppm:0.86–0.94 (m, 12H, 4CH_3_), 1.62–1.74 (m, 4H, 2CH_2_), 1.98 (s, 6H, 2CH_3_), 2.18–2.25 (m, 2H, 2CH), 3.45 (d, 4H, 2CH_2_), 3.68 (s, 6H, 2OCH_3_), 4.20–4.35 (m, 2H, 2CH), 4.65–4.70 (m, 2H, 2CH), 6.95–7.65 (m, 18H, 4Ph-H), 8.62, 9.00 (2s, 3H, pyr-H), 8.66, 8.76, 9.20 (3s, 6H, 6NH, exchangeable with D_2_O). ^13^C NMR spectrum (DMSO-d_6_), δ_C_, ppm: 15.50, 17.85, 18.24 (6C, 6 CH_3_), 23.78 (2C, 2CH), 40.26 (2C, 2CH_2_), 42.48 (2C, CH_2_), 52.76, 53.15 (4C, 4 CH), 55.72 (2C, 2OCH_3_), 114.32, 125.65, 127.28, 128.32, 128.65, 129.75, 138.68, 162.84 (24C, 4Ph-C), 131.60, 140.12, 152.20 (5C, pyr-C), 145.15 (2C, 2C=N), 164.14, 169.65 (4C, 4 CO-amide), 174.06 (2C, 2CO-hydrazone). MS (EI, 70 eV): *m/z* (%) = 980 (16) [M]^+^. Found, %: C 67.28; H 6.62; N 12.76. C_55_H_65_N_9_O_8_ (980.16). Calculated, %: C 67.40; H 6.68; N 12.86.

**N^3^,N^5^-Bis{1-[(1-(2-(1-(4-chlorophenyl)ethylidene)hydrazinyl)-4-methyl-1-oxopentan-2-yl)-amino]-1-oxo-3-phenylpropan-2-yl}pyridine-3,5-dicarboxamide (5b):** Yield 60 %, mp 184–186 °C (DMF/H_2_O). [*α*]${}_{D}^{25}$ = −99 (*c* = 0.5, DMF). IR ν, cm^−1^: 3467–3345 (NH), 3086 (CH-Ar), 2979 (CH-aliph.), 1654, 1535, 1254 (C=O, amide I, II and III). ^1^H NMR spectrum (DMSO-d_6_), δ_H_, ppm: 0.88–0.92 (m, 12H, 4CH_3_), 1.64–1.70 (m, 4H, 2CH_2_), 2.05 (s, 6H, 2CH_3_), 2.20–2.24 (m, 2H, 2CH), 3.46 (d, 4H, 2CH_2_), 4.26–4.34 (m, 2H, 2CH), 4.68–4.72 (m, 2H, 2CH), 7.10–7.90 (m, 18H, 4Ph-H), 8.65, 9.04 (2s, 3H, pyr-H), 8.70, 8.78, 9.18 (3s, 6H, 6NH, exchangeable with D_2_O). ^13^C NMR spectrum (DMSO-d_6_), δ_C_, ppm: 15.85, 17.80, 18.12 (6C, 6 CH_3_), 23.84 (2C, 2CH), 40.38 (2C, 2CH_2_), 42.50 (2C, CH_2_), 52.74, 53.10 (4C, 4 CH), 125.65, 127.28, 128.32, 128.78, 129.05, 134.95, 135.68, 136.84 (24C, 4Ph-C), 131.70, 140.18, 152.26 (5C, pyr-C), 144.98 (2C, 2C=N), 164.34, 169.65 (4C, 4 CO-amide), 173.96 (2C, 2CO-hydrazone). MS (EI, 70 eV): *m/z* (%) = 988 (24) [M-1]^+^. Found, %: C 64.24; H 5.90; Cl 7.08; N 12.65. C_53_H_59_Cl_2_N_9_O_6_ (988.99). Calculated, %: C 64.36; H 6.01; Cl 7.17; N 12.75.

**N^3^,N^5^-Bis{1-[(1-(2-(1-(4-bromophenyl)ethylidene)hydrazinyl)-4-methyl-1-oxopentan-2-yl)-amino]-1-oxo-3-phenylpropan-2-yl}pyridine-3,5-dicarboxamide (5c):** Yield 72 %, mp 210–212 °C (DMF/H_2_O). [*α*]${}_{D}^{25}$ = −124 (*c* = 0.5, DMF). IR ν, cm^−1^: 3462–3345 (NH), 3090 (CH-Ar), 2988 (CH-aliph.), 1655, 1532, 1250 (C=O, amide I, II and III). ^1^H NMR spectrum (DMSO-d_6_), δ_H_, ppm: 0.84–0.90 (m, 12H, 4CH_3_), 1.60–1.65 (m, 4H, 2CH_2_), 2.02 (s, 6H, 2CH_3_), 2.18–2.22 (m, 2H, 2CH), 3.48 (d, 4H, 2CH_2_), 4.25–4.35 (m, 2H, 2CH), 4.66–4.70 (m, 2H, 2CH), 7.14–7.82 (m, 18H, 4Ph-H), 8.62, 9.08 (2s, 3H, pyr-H), 8.72, 8.76, 9.16 (3s, 6H, 6NH, exchangeable with D_2_O). ^13^C NMR spectrum (DMSO-d_6_), δ_C_, ppm: 15.90, 17.60, 17.86 (6C, 6 CH_3_), 23.80 (2C, 2CH), 40.42 (2C, 2CH_2_), 42.56 (2C, CH_2_), 52.78, 53.15 (4C, 4 CH), 125.55, 126.04, 127.56, 128.24, 128.70, 130.75, 135.98, 136.34 (24C, 4Ph-C), 131.62, 140.26, 152.28 (5C, pyr-C), 145.08 (2C, 2C=N), 164.65, 168.98 (4C, 4 CO-amide), 174.16 (2C, 2CO-hydrazone). MS (EI, 70 eV): *m/z* (%) = 1078 (14) [M]^+^. Found, %: C 58.88; H 5.45; N 11.62. C_53_H_59_Br_2_N_9_O_6_ (1077.90). Calculated, %: C 59.06; H 5.52; N 11.69.

**N^3^,N^5^-Bis{1-[(4-methyl-1-oxo-1-(2-(1-(p-tolyl)ethylidene)hydrazinyl)pentan-2-yl)amino]-1-oxo-3-phenylpropan-2-yl}pyridine-3,5-dicarboxamide (5d):** Yield 55%, mp 235–237 °C (DMF/H_2_O). [*α*]${}_{D}^{25}$ = −78 (*c* = 0.5, DMF). IR ν, cm^−1^: 3465–3340 (NH), 3088 (CH-Ar), 2980 (CH-aliph.), 1656, 1536, 1256 (C=O, amide I, II, and III). ^1^H NMR spectrum (DMSO-d_6_), δ_H_, ppm:0.88–0.92 (m, 12H, 4CH_3_), 1.66–1.70 (m, 4H, 2CH_2_), 2.00 (s, 6H, 2CH_3_), 2.18–2.24 (m, 2H, 2CH), 2.34 (s, 6H, 2CH_3_), 3.43 (d, 4H, 2CH_2_), 4.23–4.30 (m, 2H, 2CH), 4.66–4.72 (m, 2H, 2CH), 7.12–7.68 (m, 18H, 4Ph-H), 8.65, 9.05 (2s, 3H, pyr-H), 8.68, 8.75, 9.18 (3s, 6H, 6NH, exchangeable with D_2_O). ^13^C NMR spectrum (DMSO-d_6_), δ_C_, ppm: 16.80, 17.90, 18.15, 21.12 (8C, 8 CH_3_), 23.86 (2C, 2CH), 40.35 (2C, 2CH_2_), 42.52 (2C, CH_2_), 52.75, 53.18 (4C, 4 CH), 125.72, 126.45, 127.35, 128.40, 128.92, 134.08, 136.10, 139.56 (24C, 4Ph-C), 131.68, 140.25, 152.25 (5C, pyr-C), 145.60 (2C, 2C=N), 164.12, 169.68 (4C, 4 CO-amide), 174.18 (2C, 2CO-hydrazone). MS (EI, 70 eV): *m/z* (%) = 948 (42) [M]^+^. Found, %: C, 69.60; H, 6.86; N, 13.22. C_55_H_65_N_9_O_6_ (948.18). Calculated, %: C, 69.67; H, 6.91; N, 13.30.

**N^3^,N^5^-Bis{1-[(4-methyl-1-(2-(1-(4-nitrophenyl)ethylidene)hydrazinyl)-1-oxopentan-2-yl)-amino]-1-oxo-3-phenylpropan-2-yl}pyridine-3,5-dicarboxamide (5e).** Yield 72 %, mp 256–258 °C (DMF/H_2_O). [*α*]${}_{D}^{25}$ = −104 (*c* = 0.5, DMF). IR ν, cm^−1^: 3485–3354 (NH), 3092 (CH-Ar), 2986 (CH-aliph.), 1655, 1533, 1255 (C=O, amide I, II, and III). ^1^H NMR spectrum (DMSO-d_6_), δ_H_, ppm: 0.86–0.90 (m, 12H, 4CH_3_), 1.66–1.74 (m, 4H, 2CH_2_), 2.10 (s, 6H, 2CH_3_), 2.24–2.26 (m, 2H, 2CH), 3.45 (d, 4H, 2CH_2_), 4.25–4.32 (m, 2H, 2CH), 4.65–4.68 (m, 2H, 2CH), 7.08–7.26 (m, 10H, 2Ph-H), 8.00–8.28 (m, 8H, 2Ph-H), 8.58, 9.03 (2s, 3H, pyr-H), 8.65, 8.75, 9.15 (3s, 6H, 6NH, exchangeable with D_2_O). ^13^C NMR spectrum (DMSO-d_6_), δ_C_, ppm: 16.04, 17.88, 18.06 (6C, 6 CH_3_), 23.95 (2C, 2CH), 40.45 (2C, 2CH_2_), 42.54 (2C, CH_2_), 52.73, 53.13 (4C, 4 CH), 125.63, 126.86, 127.32, 127.90, 128.24, 136.70, 143.06, 149.85 (24C, 4Ph-C), 131.55, 140.25, 152.20 (5C, pyr-C), 145.00 (2C, 2C=N), 163.98, 169.40 (4C, 4 CO-amide), 174.34 (2C, 2CO-hydrazone). MS (EI, 70 eV): *m/z* (%) = 1010 (8) [M-1]^+^. Found, %: C, 62.94; H, 5.80; N, 15.20. C_53_H_59_N_11_O_10_ (1010.12). Calculated, %: C, 63.02; H, 5.89; N, 15.25.

**N^3^,N^5^-bis{1-[(1-(2-(furan-2-ylmethylene)hydrazinyl)-4-methyl-1-oxopentan-2-yl)amino]-1-oxo-3-phenylpropan-2-yl}pyridine-3,5-dicarboxamide (6a):**Yield 75%, mp 205–207 °C (DMF/Ether). [*α*]${}_{D}^{25}$ = −105 (*c* = 0.5, DMF). - IR (film): ν = 3495–3350 (NH), 3095 (CH-Ar), 2985 (CH-aliph.), 1654, 1532, 1250 (C=O, amide I, II, and III) cm^−1^.^1^H NMR spectrum (DMSO-d_6_), δ_H_, ppm: 0.85–0.92 (m, 12H, 4CH_3_), 1.68–1.74 (m, 4H, 2CH_2_), 2.22–2.34 (m, 2H, 2CH), 3.42 (d, 4H, 2CH_2_), 4.16–4.26 (m, 2H, 2CH), 4.45–4.58 (m, 2H, 2CH), 6.90–6.90 (m, 6H, 2 furyl ring), 7.12–7.38 (m, 10H, 2 Ph-H), 7.98 (s, 2H, 2CH=N), 8.32, 9.10 (2s, 3H, pyr-H), 8.78, 8.80, 9.16 (3s, 6H, 6NH, exchangeable with D_2_O). ^13^C NMR spectrum (DMSO-d_6_), δ_C_, ppm: 18.60, 19.15 (4C, 4 CH_3_), 22.96 (2C, 2CH), 40.25 (2C, 2CH_2_), 42.48 (2C, CH_2_), 52.56, 53.34 (4C, 4 CH), 112.05, 117.95, 143.45, 148.16 (8C, 2 furyl-C), 125.84, 127.42, 127.98, 137.85 (12C, 2Ph-C), 131.62, 140.34, 152.40 (5C, pyr-C), 137.12 (2C, 2C=N), 163.95, 169.48 (4C, 4 CO-amide), 178.46 (2C, 2CO-hydrazone). MS (EI, 70 eV): *m/z* (%) = 872 (16) [M]^+^. Found, %: C, 64.65; H, 6.10; N, 14.39. C_47_H_53_N_9_O_8_ (872.00). Calculated, %: C, 64.74; H, 6.13; N, 14.46.

**N^3^,N^5^-bis{1-[(4-methyl-1-oxo-1-(2-(thiophen-2-ylmethylene)hydrazinyl)pentan-2-yl)amino]-1-oxo-3-phenylpropan-2-yl}pyridine-3,5-dicarboxamide (6b).** Yield 70%, mp 218–220 °C (DMF/Ether). [*α*]${}_{D}^{25}$ = −110 (*c* = 0.5, DMF). IR (film): ν = 3482–3365 (NH), 3084 (CH-Ar), 2978 (CH-aliph.), 1655, 1535, 1254 (C=O, amide I, II, and III) cm^−1^.^1^H NMR spectrum (DMSO-d_6_), δ_H_, ppm: 0.82–0.90 (m, 12H, 4CH_3_), 1.65–1.70 (m, 4H, 2CH_2_), 2.20–2.30 (m, 2H, 2CH), 3.44 (d, 4H, 2CH_2_), 4.18–4.30 (m, 2H, 2CH), 4.46–4.55 (m, 2H, 2CH), 7.18–7.56 (m, 16H, 2 Ph-H + 2 furyl ring), 7.86 (s, 2H, 2CH=N), 8.36, 9.08 (2s, 3H, pyr-H), 8.75, 8.84, 9.18 (3s, 6H, 6NH, exchangeable with D_2_O). ^13^C NMR spectrum (DMSO-d_6_), δ_C_, ppm: 18.32, 19.10 (4C, 4 CH_3_), 22.78 (2C, 2CH), 40.55 (2C, 2CH_2_), 42.68 (2C, CH_2_), 52.54, 53.32 (4C, 4 CH), 126.45, 127.90, 130.45, 144.06 (8C, 2 thienyl-C), 125.80, 127.52, 128.14, 137.80 (12C, 2Ph-C), 131.65, 140.36, 152.42 (5C, pyr-C), 125.18 (2C, 2C=N), 163.86, 169.45 (4C, 4 CO-amide), 178.42 (2C, 2CO-hydrazone). MS (EI, 70 eV): *m/z* (%) = 904 (24) [M]^+^. Found, %: C, 62.32; H, 5.86; N, 13.88; S, 7.00. C_47_H_53_N_9_O_6_S_2_ (904.12). Calculated, %: C, 62.44; H, 5.91; N, 13.94; S, 7.09.

### 3.2. Biological Evaluation

#### 3.2.1. In Vitro Cytotoxic Activity against MCF-7 Cancer Cells 

Human breast cancer (MCF-7) cells, obtained from Sigma-Aldrich Chemie GmbH, Taufkirchen, Germany, were propagated in RPMI-1640 supplemented with 10% heat inactivated FBS, 2 mM L-glutamine, and 1% standard antibiotic solution. Cells were incubated in a 5% CO_2_humidified incubator at 37 °C and passaged bi-weekly. The in vitro anti-proliferative activity of the newly synthesized derivatives was assayed using the standard MTT technique [54,55,56].

The results were expressed as IC_50_. Experiments were repeated at least in triplicate, to obtain good reproducibility between replicate wells with standard errors below 10%. Furthermore, the cytotoxic effects of the prepared derivatives were evaluated against normal nonmalignant cells, non-tumorigenic MCF-10A, in order to find out if the synthesized derivatives have toxicity against normal cells. Additionally, the results were compared with reference compounds (cisplatin and milaplatin) as positive controls.

#### 3.2.2. In Vivo Human Breast Cancer Xenograft 

The breast cancer xenograft model protocol was approved by the Institutional Animal Use and Care Committee of the University of Alabama at Birmingham (50-01-05-08B). Female athymic pathogen-free nude mice (nu/nu, 4–6 weeks) were used. Firstly, MCF-7 xenografts were initiated by implanting pellets slowly releasing estrogen for two months (1.7mg 17β-estradiol/pellet) subcutaneously in the female nude mice. After 24 h, confluent MCF-7 cells were harvested, washed two times with serum-free medium, re-suspended, and injected subcutaneously (s.c.) (5 × 10^6^ cells, total volume 0.2 mL) into the left inguinal area of the mice. Caliper measurement was used to measure tumor growth in two perpendicular diameters of the implant after 48 h, and its volume was determined. Mice grafted with MCF-7 were divided into different groups (7–10 mice/group). Untreated mice received the solvent only. Treated groups received different prepared derivatives as previously described [57].

#### 3.2.3. In Vitro and In Vivo p53 Ubiquitination

Different prepared derivatives **4**–**6** were evaluated for their inhibitory potential against p53 ubiquitination according to the previously reported procedure [57].

#### 3.2.4. LDHA Inhibition Assay

Lactate dehydrogenase assay kit (Abcam, ab102526) and recombinant human LDHA protein (Abcam, ab93699) were used to assess the inhibitory effect of the tested compounds. First, we generated an NADH standard curve for colorimetric detection by measuring the OD (450 nm) at different molar amounts of NADH. The linear regression equation of the curve was derivatized in Graphpad Prism. Tested compounds were dissolved in DMSO and their inhibitory activity was assessed at 140 and 300 μM strength. Galloflavin (Sigma, SML0776) at the same molar concentration was used as a known LDH inhibitor. The master reaction mix composed of LDH assay buffer and 10 ng human LDHA substrate per reaction was prepared. Tested compounds were added and the final reaction volume was adjusted to 50 μL. Absorbance at 450 nm was taken after 2–3 min (T-initial) and continued every minute for 118 min (T-final). The change in measurement over time (delta A450) was calculated as T-final minus T-initial. The amount of NADH generated by the assay between T-initial and T-final was deduced by comparing the A450 of each sample to the standard NADH standard curve. LDHA activity was calculated in milliunits per mL as the amount of NADH generated by the assay / (reaction time in minutes (118) x reaction volume in mL (0.005) [45].

### 3.3. Molecular Docking Studies

The three-dimensional X-ray structure of LDHA (PDB code: 4ZVV) [53] was chosen as the template for the modeling study of the screened compounds **4**–**6**. The crystal structure was derived from the RCSB Protein Data Bank and the molecular docking procedure was performed using MOE, 10.2008 software following the reported procedure [51,52].

## 4. Conclusions

In conclusion, a series of new tetrapeptide Schiff bases **3**–**6**were designed, synthesized, and evaluated for their antitumor activities against breast MCF-7 cell lines. These compounds exhibited potent antiproliferative activities in comparison with the standards, cisplatin and milaplatin. All of these derivatives were subjected to evaluation ofin vitroandin vivosuppression of p53 ubiquitination and inhibition assay for LDHA enzyme. Molecular docking study was further performed to illustrate the possible binding interactions with LDHA that might be a promising base for the further development of novel anticancer agents with excellent LDH inhibitory activity.

## Figures and Tables

**Table 1:** Molecular docking data of the synthesized compounds **4**–**6** in comparison with GNE-140 within the active site of LDHA kinase using MOE software version 2008.10.

Compd. NO.	Docking Score (Kcal/mol)	Amino Acid Residues (Bond Length A^o^)	Atoms of Compound	Type of bond
GNE-140	−9.73	Asn137(2.75); Arg168(2.99); His192(2.74)	O(OH); O(CO); O(OH)	H- acc H- acc H-acc
4a	−14.22	Asn137(2.54); Arg168; His192(2.66)	O(CONH); 2-pridyl; N(2-pridyl)	H- acc Arene-cation H-acc
4b	−13.62	Asn137(2.68); Arg168; His192(2.88)	O(CONH); 3-pridyl; N(3-pridyl)	H- acc Arene-cation H-acc
4c	−11.29	Asn137(2.77); Arg168	O(CONH); 3-pridyl	H- acc Arene-cation
5a	−13.01	Arg98; Arg168; Tyr238(3.20); Thr247(3.00)	C_6_H_5_-4-CH_3_; C_6_H_5_-4-CH_3_; O(CONH); O(CONH)	Arene-cation Arene-cation H-acc H- acc
5b	−12.78	Arg168; His192(2.90) Tyr238(3.00);	C_6_H_5_-4-OCH_3_; O(C_6_H_5_-4-OCH_3_); O(CONH)	Arene-cation H-acc H- acc
5c	−15.60	Arg98; Arg168; Tyr238(3.06); Thr247(2.70)	C_6_H_5_-4-Cl; C_6_H_5_-4-Cl; O(CONH);O(CONH)	Arene-cation Arene-cation H-accH- acc
5d	−11.89	Arg98; Arg168; Asn137(2.88)	C_6_H_5_-4-Br; C_6_H_5_-4-Br; O(CONH)	Arene-cation Arene-cation H-acc
5e	−11.37	Arg168; Asn137(3.05)	C_6_H_5_-4-NO_2_; O(CONH)	Arene-cation H-acc
6a	−12.06	Asn137(2.88); Tyr238	O(CONH); furan	H- acc Arene-cation
6b	−12.88	Asn137(2.35); Tyr238	O(CONH); thiophene	H- acc Arene-cation

## References

[1] Marqus S., Pirogova E., Piva T.J. (2017). Evaluation of the use of therapeutic peptides for cancer treatment. J. Biomed. Sci..

[2] Chakrabarti S., Jahandideh F., Wu J. (2014). Food-derived bioactive peptides on inflammation and oxidative stress. Biomed. Res. Int..

[3] Yang R.Y., Zhang Z.F., Pei X.R., Han X.L., Wang J.B., Wang L.L., Long Z., Shen X.Y., Li Y. (2009). Immunomodulatory effects of marine oligopeptide preparation from Chum Salmon (Oncorhynchus keta) in mice. Food Chem..

[4] Cicero A.F.G., Fogacci F., Colletti A. (2017). Potential role of bioactive peptides in prevention and treatment of chronic diseases: A narrative review. Br. J. Pharmacol..

[5] Holohan C., Van Schaeybroeck S., Longley D.B., Johnston P.G. (2013). Cancer drug resistance: an evolving paradigm. Nat. Rev. Cancer.

[6] Boohaker R.J., Lee M.W., Vishnubhotla P., Perez J.M., Khaled A.R. (2012). The use of therapeutic peptides to target and to kill cancer cells. Curr. Med. Chem..

[7] Gautam A., Kapoor P., Chaudhary K., Kumar R., Raghava G.P. (2014). Tumor homing peptides as molecular probes for cancer therapeutics, diagnostics and theranostics. Curr. Med. Chem..

[8] Vlieghe P., Lisowski V., Martinez J., Khrestchatisky M. (2010). Synthetic therapeutic peptides: science and market. Drug Discov. Today.

[9] Blanco-Míguez A., Gutiérrez-Jácome A., Pérez-Pérez M., Pérez-Rodríguez G., Catalán-García S., Fdez-Riverola F., Lourenço A., Sánchez B. (2016). From amino acid sequence to bioactivity: scientific evidence on antitumor peptides. Protein Sci..

[10] Lopci E., Nanni C., Rampin L., Rubello D., Fanti S. (2008). Clinical applications of 68Ga-1239 DOTANOC in neuroendocrine tumours. Minerva Endocrinol..

[11] Emons G., Sindermann H., Engel J., Schally A.V., Grundker C. (2009). Luteinizing hormone-releasing hormone receptor-targeted chemotherapy using AN-152. Neuroendocrinology.

[12] Amr A.E., Naglah A.M., Sabry N.M., Ibrahim A.A., Elsayed E.A., Attar A. (2019). Synthesis and investigation of 3,5-bis-linear and macrocyclic tripeptidopyridine candidates by using l-valine, N,N′-(3,5-pyridinediyldicarbonyl)bis-dimethyl ester as synthon. Z. Naturforsch..

[13] Khalifa N.M., Naglah A.M., Al-Omar M.A., Abo-Ghalia M.H., Amr A.E. (2014). Synthesis and reactions of new chiral linear carboxamides with an incorporated peptide linkage using nalidixic acid and amino acids as starting materials. Z. Naturforsch..

[14] Khayyat S., Amr A.E. (2014). Synthesis and biological activities of some new (Nα-dinicotinoyl)-bis- L-leucyllnear and macrocyclic peptides. Molecules.

[15] Amr A.E., Abu Ghalia M.H., Al-Omar M.A., Abdalla M.M. (2016). Anticancer activities of some macrocyclic peptidocalix[4]arene and peptidopyridine candidates. Lat. Am. J. Pharm..

[16] Fahmi N., Shrivastava S., Meena R., Joshi S., Singh R. (2013). Microwave assisted synthesis, spectroscopic characterization and biological aspects of some new chromium (III) complexes derived from NΑ O donor Schiff bases. N. J. Chem..

[17] Baseer M.A., Jadhav V.D., Phule R.M., Archana Y.V., Vibhute Y.B. (2000). Synthesis and antibacterial activity of some new Schiff bases. Orient J. Chem..

[18] Singh W.M., Dash B.C. (1988). Synthesis of some new Schiff bases containing thiazole and oxazole nuclei and their fungicidal activity. Pesticides.

[19] Sridhar S., Pandeya S., De Clercq E. (2000). Synthesis and anti-HIV activity of some isatin derivatives. Boll. Chim. Farm..

[20] Das B.P., Choudhury T.R., Das G.K., Chowdhury D.N., Choudhury B. (1994). Comparative studies on largicidal activity of some Schiff bases with correspondian amines. Chem. Environ. Res..

[21] Sparatore F., Pirisino G., Alamanni M., Manca-Dimich P., Satta M. (1978). Azomethine derivatives with anti-inflammatory activity. Boll. Chim. Farm..

[22] Chaviara A.T., Christidis P.C., Papageorgiou A., Chrysogelou E., Hadjipavlou-Litina D.J., Bolos C.A. (2005). In vivo anticancer, anti-inflammatory, and toxicity studies of mixed-ligand Cu(II) complexes of dien and its Schiff dibases with heterocyclic aldehydes and 2-amino-2-thiazoline. Crystal structure of [Cu (dien)(Br)(2a-2tzn)](Br)(H(2)O). J. Inorg. Biochem..

[23] Jamshidvand A., Sahihi M., Mirkhani V., Moghadam M., Mohammadpoor-Baltork I., Tangestaninejad S., Rudbari H.A., Kargar H., Keshavarzi R., Gharaghani S. (2018). Studies on DNA binding properties of new Schiff base ligands using spectroscopic, electrochemical and computationalmethods: Influence of substitutions on DNA-binding. J. Mol. Liq..

[24] Hameeda A., Al-Rashida M., Uroosc M., Ali S.A., Khan K.M. (2017). Schiff bases in medicinal chemistry: A patent review (2010–2015). Expert. Opin. Ther. Pat..

[25] Di Stefano G., Manerba M., Di Ianni L., Fiume L. (2016). Lactate dehydrogenase inhibition: Exploring possible applications beyond cancer treatment. Future Med. Chem..

[26] Warburg O. (1956). On the origin of cancer cells. Science.

[27] Talaiezadeh A., Shahriari A., Tabandeh M.R., Fathizadeh P., Mansouri S. (2015). Kinetic characterization of lactate dehydrogenase in normal and malignant human breast tissues. Cancer Cell Int..

[28] Ward R.A., Brassington C., Breeze A.L., Caputo A., Critchlow S., Davies G., Goodwin L., Hassall G., Greenwood R., Holdgate G.A. (2012). Design and synthesis of novel lactate dehydrogenase A inhibitors by fragment-based lead generation. J. Med. Chem..

[29] Kohlmann A., Zech S.G., Li F., Zhou T., Squillace R.M., Commodore L., Greenfield M.T., Lu X., Miller D.P., Huang W.S. (2013). Fragment growing and linking lead to novel nanomolar lactate dehydrogenase inhibitors. J. Med. Chem..

[30] Altamimi A.S., Alafeefy A.M., Balode A., Vozny I., Pustenko A., El Shikh M.E., Alasmary F.A.S., Abdel-Gawad S.A., Žalubovskis R. (2018). Symmetric molecules with 1,4-triazole moieties as potent inhibitors of tumour-associated lactate dehydrogenase-A. J. Enzy. Inh. Med. Chem..

[31] Amr A.E., Abo-Ghalia M.H., Abdalla M.M. (2006). Synthesis of novel macrocyclic peptidocalix[4]arenes and peptido-pyridines as precursors for potential molecular metallacages, chemo-sensors and biologically active candidates. Z. Naturforsch..

[32] Amr A.E., Abo-Ghalia M.H., Moustafa G.O., Al-Omar M.A., Nossier E.S., Elsayed E.A. (2018). Synthesis and characterization of some newly macrocyclic pentapeptide derivatives as anticancer activity. Molecules.

[33] Amr A.E., Al-Omar M.A., Abdalla M.M. (2016). Analgesic, anti-convulsant and antiparkinsonian activities of some synthesized 2,6-bis(tetracarbox-amide)-pyridine and macrocyclic tripeptide derivatives. Int. J. Pharmacol..

[34] Khayyat S., Amr A.E., Abd El-Salam O.I., Al-Omar M.A., Abdalla M.M. (2015). Analgesic and anti-inflammatory activities of some newly synthesized 3,5-bis[(peptidohydrazinyl)pyridine Schiff bases. Int. J. of Pharmacol..

[35] Flefel E.M., Alsafi M.A., Alahmadi S.M., Amr A.E., Fayed A.A. (2018). Antimicrobial activities of some synthesized macrocyclic pentaazapyridine and dipeptide pyridine derivatives. Biomed. Res..

[36] Khalifa N.M., Amr A.E., Al-Omar M.A., Nossier E.S. (2016). Synthesis, characterization, and antimicrobial activity of some chiral linear carboxamides with incorporated peptide linkage. Russ. J. General Chem..

[37] Azab M.E., Flefel E.M., Sabry N.M., Amr A.E. (2016). Synthesis and antimicrobial activity of some linear dipeptide pyridine and macrocyclic pentaazapyridine candidates. Z. Naturforsch..

[38] Amr A.E., Ali K.A., Abdalla M.M. (2009). Cytotoxic, antioxidant activities and structure activity relationship of some newly synthesized terpenoidaloxaliplatin analogs. Eur. J. Med. Chem..

[39] Naglah A.M., Amr A.E., Abdel Mageid R.E., Al-Omar M.A., Abd El-Salam O.I. (2019). Synthesis of chiral 3,5-bis(L-phenylalaninyl-L-leucinyl)pyridine Schiff base and their macrocyclic carboxaimide derivatives using 3,5-bis(L-phenylalaninyl)pyridine methyl ester. Z. Naturforsch..

[40] Abo-Ghalia M., Amr A. (2004). Synthesis and investigation of a new cyclo (*N^α^*-dipicolinoyl) pentapeptide of a breast and CNS cytotoxic activity and an ionophoric specificity. Amino Acids.

[41] AbouMelha K.S.A., Al-Hazmi G.A.A., Refat M.S. (2017). Synthesis of nano-metric gold complexes with new Schiff bases derived from 4-aminoantipyrene, their structures and anticancer activity. Russ. J. Gen. Chem..

[42] Padhye S., Yang H., Jamadar A., Cui Q.C., Chavan D., Dominiak K., McKinney J., Banerjee S., Dou Q.P., Sarkar F.H. (2009). New difluoroKnoevenagel condensates of curcumin, their Schiff bases and copper complexes as proteasome inhibitors and apoptosis inducers in cancer cells. Pharm. Res..

[43] Dai M.-S., Lu H. (2004). Inhibition of MDM2-mediated p53 Ubiquitination and Degradation by Ribosomal Protein L5. J. Biol. Chem..

[44] Manerba M., Vettraino M., Fiume L., Di Stefano G., Sartini A., Giacomini E., Buonfiglio R., Roberti M., Recanatini M. (2012). Galloflavin (CAS 568-80-9): A novel inhibitor of lactate dehydrogenase. Chem. Med. Chem..

[45] Amr A.E., El-Shehry M.F., Ibrahim A.A., Hosni H.M., Al-Omar M.A., Ghabbour H.A. (2019). Synthesis and molecular docking of new thiophene derivatives as lactate dehydrogenase-A inhibitors. Mini Rev. in Med. Chem..

[46] Farabegoli F., Vettraino M., Manerba M., Fiume L., Roberti M., Di Stefano G. (2012). Galloflavin, a new lactate dehydrogenase inhibitor, induces the death of human breast cancer cells with different glycolytic attitude by affecting distinct signaling pathways. Eur. J. Pharm. Sci..

[47] Vettraino M., Manerba M., Govoni M., Di Stefano G. (2013). Galloflavin suppresses lactate dehydrogenase activity and causes MYC downregulation in Burkitt lymphoma cells through NAD/NADH-dependent inhibition of sirtuin-1. Anticancer Drugs.

[48] Manerba M., Di Ianni L., Fiume L., Roberti M., Recanatini M., Di Stefano G. (2015). Lactate dehydrogenase inhibitors sensitize lymphoma cells to cisplatin without enhancing the drug effects on immortalized normal lymphocytes. Eur. J. Pharm. Sci..

[49] Han X., Sheng X., Jones H.M., Jackson A.L., Kilgore J., Stine J.E., Schointuch M.N., Zhou C., Bae-Jump V.L. (2015). Evaluation of the anti-tumor effects of lactate dehydrogenase inhibitor galloflavin in endometrial cancer cells. J. Hematol. Oncol..

[50] Manerba M., Di Ianni L., Govoni M. (2017). Lactate dehydrogenase inhibitors can reverse inflammation induced changes in colon cancer cells. Eur. J. Pharm. Sci..

[51] Elzahabi H.S.A., Nossier E.S., Khalifa N.M., Alasfoury R.A., El-Manawat M.A. (2018). Anticancer evaluation and molecular modeling of multi-targeted kinase inhibitors based pyrido[2–d]pyrimidine scaffold. J. Enzy. Inh. Med. Chem..

[52] Othman I.M.M., Gad-Elkareem M.A.M., El-Naggar M., Nossier E.S., Amr A.E. (2019). Novel phthalimide based analogues: Design, synthesis, biological evaluation, and molecular docking studies. J. Enzy. Inh. Med. Chem..

[53] Boudreau A., Purkey H.E., Hitz A., Robarge K., Peterson D., Labadie S., Kwong M., Hong R., Gao M., Del Nagro C. (2016). Metabolic plasticity underpins innate and acquired resistance to LDHA inhibition. Nat. Chem. Biol..

[54] Elsayed E.A., Sharaf-Eldin M.A., El-Enshasy H.A., Wadaan M. (2016). In vitroassessment of anticancer properties of *Moringaperegrina* essential seed oil on different cell lines. Pak. J. Zool..

[55] Elsayed E.A., Farooq M., Dailin D., El-Enshasy H.A., Othman N.Z., Malek R., Danial E., Wadaan M. (2017). In vitro and in vivo biological screening of kefiran polysaccharide produced by *Lactobacillus kefiranofaciens*. Biomed. Res..

[56] Amr A.E., El-Naggar M., Al-Omar M.A., Elsayed E.A., Abdalla M.M. (2018). In vitro and in vivo anti-breast cancer activities of some synthesized pyrazolinyl-estran-17-one candidates. Molecules.

[57] Amr A.E., Elsayed E.A., Al-Omar M.A., BadrEldin H.O., Nossier E.S., Abdallah M.M. (2019). Design, Synthesis, Anticancer Evaluation and Molecular Modeling of Novel Estrogen Derivatives. Molecules.

